# Near-lifespan mesoscopic optical imaging of cerebrovascular function reveals age and sex differences in preclinical Alzheimer’s disease model

**DOI:** 10.1093/braincomms/fcaf472

**Published:** 2025-12-03

**Authors:** Noah Schweitzer, Christopher Cover, Howard Aizenstein, Minjie Wu, Alberto Vazquez, Bistra Iordanova

**Affiliations:** Department of Bioengineering, University of Pittsburgh, Pittsburgh, PA 15213, United States of America; Department of Bioengineering, University of Pittsburgh, Pittsburgh, PA 15213, United States of America; Department of Bioengineering, University of Pittsburgh, Pittsburgh, PA 15213, United States of America; Department of Psychiatry, University of Pittsburgh School of Medicine, Pittsburgh, PA 15213, United States of America; Department of Psychiatry, University of Pittsburgh School of Medicine, Pittsburgh, PA 15213, United States of America; Department of Bioengineering, University of Pittsburgh, Pittsburgh, PA 15213, United States of America; Department of Radiology, University of Pittsburgh School of Medicine, Pittsburgh, PA 15213, United States of America; Department of Bioengineering, University of Pittsburgh, Pittsburgh, PA 15213, United States of America

**Keywords:** Alzheimer’s disease, vascular dysfunction, neurovascular coupling, cerebrovascular reactivity, sex differences

## Abstract

Growing evidence suggests vascular dysfunction plays a critical role in the early stages of Alzheimer’s disease, commonly associated with amyloid-β deposition. This vascular dysfunction is particularly relevant in the context of cerebral amyloid angiopathy, where amyloid-β accumulates within cerebral vessel walls. Notably, sex differences impact progression of both Alzheimer’s disease and cerebrovascular dysfunction, with post-menopausal females displaying increased small vessel disease burden and diminished carbon dioxide reactivity compared to older males and pre-menopausal females. Moreover, the cerebrovasculature is a target of sex hormones where they exert influence in numerous vascular functions and pathologies across lifespan. Combined, cerebrovascular dysfunction along with amyloid-β deposition may have differential effects on sex. Despite observational studies in humans, preclinical mechanistic and functional research on sex-specific vascular differences in Alzheimer’s disease has been limited. In this near-lifespan longitudinal study, we investigated age and sex-specific neurovascular coupling and carbon dioxide reactivity in a transgenic mouse model expressing chimeric mouse/human amyloid precursor and mutant human presenilin 1 (APP/PS1) and control mice using widefield optical imaging. Neurovascular coupling was probed via whisker stimulation and then vascular reactivity was measured using hypercapnic challenge. During whisker stimulation, neuronal activity was measured through GCaMP6f fluorescence change, while vascular response was quantified via haemoglobin-based optical intrinsic signal. Carbon dioxide reactivity was evaluated by measuring dilatory changes of vessel diameters across the cerebrovascular tree. *In vivo* two-photon microscopy was used to longitudinally measure cerebral amyloid angiopathy vessel coverage and amyloid-β tissue plaque volume. We observed that APP/PS1 mice exhibited attenuated neurovascular coupling during whisker stimulation and this response worsened through lifespan compared to controls. Compared to controls, APP/PS1 mice exhibited decreased carbon dioxide reactivity with age. No sex differences between control mice were observed in the neurovascular response to whisker, whereas during hypercapnia, control females had higher carbon dioxide reactivity than control males. While both APP/PS1 males and females showed reduced dilatory responses with age, APP/PS1 females exhibited this decrease in small arteries, whereas APP/PS1 males experienced decreased dilation in larger arteries. Diminished vascular reactivity in APP/PS1 mice was associated with increased cerebral amyloid angiopathy and amyloid-plaque burden. This study highlights sex-specific pathophysiology’s of vascular dysfunction across the lifespan. Our findings underscore needs to incorporate sex differences in preclinical Alzheimer’s disease research, given the rising importance of vascular contributions to cognitive impairment and dementia. Our findings have important implications for developing targeted, age and sex-specific biomarkers and therapeutics for cerebrovascular health in Alzheimer’s disease.

## Teaser

Schweitzer *et al*. examined how age and sex influence cerebrovascular dysfunction across the lifespan in an Alzheimer’s disease mouse model. Using optical imaging, they found that neurovascular coupling and vascular reactivity decline with age, with females showing small-vessel deficits and males showing large-vessel dysfunction, underscoring the importance of sex-specific approaches in Alzheimer’s research.

## Graphical Abstract

**Graphical Abstract:**
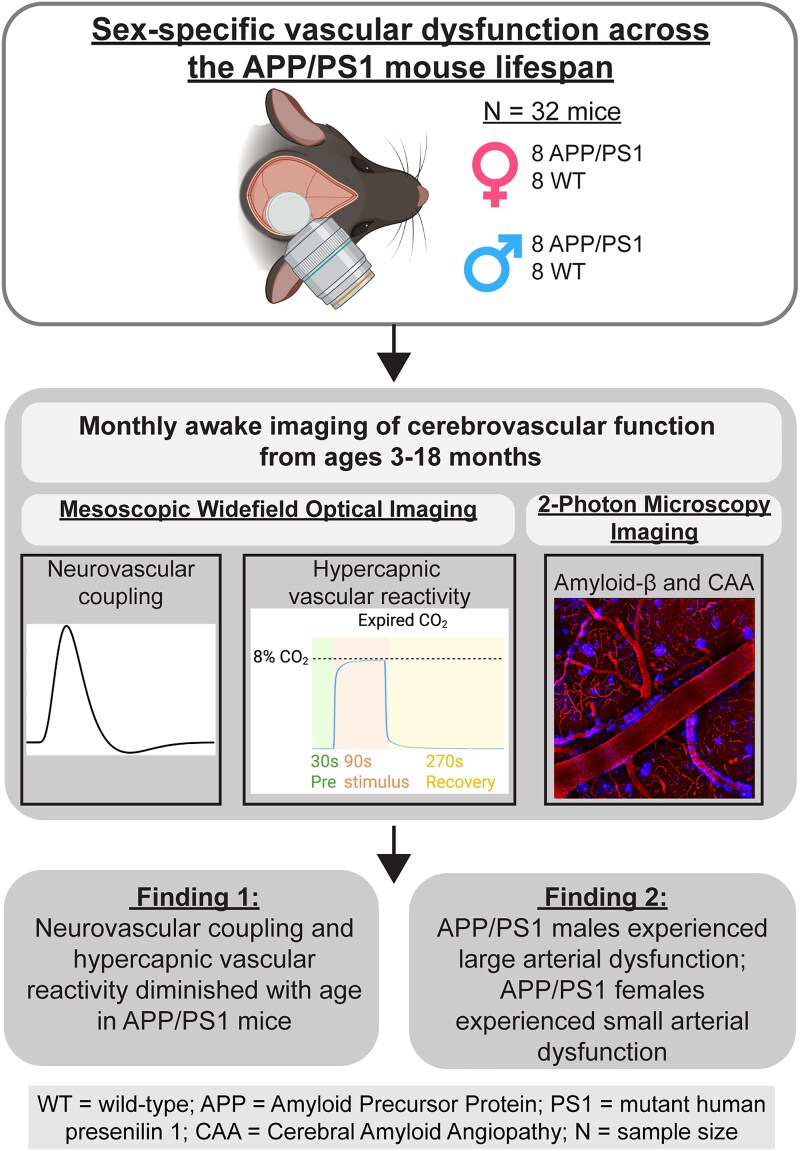

## Introduction

Alzheimer’s disease (AD), the most common cause of dementia, has become a major public health burden as populations continue to age. Amyloid β (Aβ) plaques are a pathological hallmark of AD and accumulate before the initial onset of cognitive decline.^1^ In the past decade, increasing evidence has demonstrated that vascular dysfunction emerges early in the progression of AD.^2,3^ This is further evidenced by cerebral amyloid angiopathy (CAA), a common pathologic feature of AD where Aβ deposits within the walls of leptomeningeal and cortical penetrating arteries.^4^ Moreover, vascular dysfunction in humans appears early in sporadic AD, as indicated by different vascular neuroimaging biomarkers, including cerebrovascular reactivity^5,6^ and resting cerebral blood flow.^2,7^ Neurovascular coupling relies on a sequence of highly coordinated multicellular events to assure a proper blood supply to activated brain areas.^8^ This tight link between neuronal signalling and cerebral blood flow presents a challenge for independent assessment of haemodynamic reserve separate from the effects of neuronal function, particularly in neurodegenerative diseases. Cerebrovascular reactivity induced by hypercapnia offers a measure independent of neuronal activity by measuring autoregulatory vasodilatory response to altered carbon dioxide concentration. This approach is becoming an increasingly popular biomarker of vascular health in humans and rodents as it allows researchers to investigate vascular function without direct neuronal control.^9^ A greater understanding of the underlying pathophysiology of how measures of cerebrovascular reactivity interact with Aβ deposition is needed to enhance its diagnostic utility.

Sex is an important factor in the risk and progression of AD, particularly in the context of vascular ageing. Roughly two-thirds of the individuals diagnosed with AD in the United States are females, a disparity that cannot be fully explained by their longer life expectancy.^10^ Although cerebrovascular dysfunction is evident in both males and females with AD, its pathways, severity and presentation appear to be sex-specific. For example, females with AD exhibit a higher prevalence of hypertension and comorbidity with cerebral small vessel disease. Males with AD are more likely to have a history of diabetes and ischaemic stroke, and AD males have a higher comorbidity with coronary artery disease.^11,12^ The observed sex differences have implicated a role of sex steroid hormones. Post-menopausal females compared to older males and pre-menopausal females have increased severity of cerebral small vessel disease burden^13-16^ and diminished cerebrovascular reactivity.^17-20^ Beyond the effects of sex steroid hormones on vasculature, sex differences in neuroinflammation, metabolism and other key molecular pathways contributing to cerebrovascular dysfunction in AD stem from both independent and interactive influences of sex chromosomes.^21^ Despite these observations in humans, preclinical rodent research has been slow to capitalize on its access to cellular resolution and identify mechanistic and functional drivers of sex differences in AD progression and the associated vascular dysfunction. Animal models of AD enable direct probing of neurovascular coupling and cerebrovascular health in the presence of differentially quantified Aβ and CAA across the full lifespan. As a result, these models can potentially inform on specific cellular and functional diagnostic biomarkers and provide the basis for targeted therapeutic approaches for all patients.

The distinct effects of sex hormones, particularly oestrogens and androgens, on the brain and its vasculature during ageing has garnered increased attention in recent years. The cerebrovasculature is a target of many sex hormones where they exert substantial influence in numerous vascular functions. Dysregulation of these hormones have been linked to many vascular pathologies across the lifespan.^22^ For instance, studies report oestrogen improves vascular tone through the activation of endothelial nitric oxide synthase,^23^ prostacyclin^24^ and endothelium-derived hyperpolarizing factor,^25^ all of which act on smooth muscle cells to induce relaxation.^26^ In addition to intracellular transcriptional control of vasoactive molecules, estrogenic also binds directly to rapid-signalling membrane receptors on the surface of vascular cells to induce vasodilation.^27^ The vasodilator effects of oestrogen are thought to contribute to the higher cerebrovascular reactivity observed in healthy females compared to healthy males.^28-30^ The beneficial vascular effects of the oestrogens are profoundly affected by ageing, however the progression of these functional changes across the lifespan remains poorly understood.^31^ Cerebrovascular reactivity in humans is typically measured using global blood-oxygen-level-dependent (BOLD) functional MRI (fMRI), or transcranial doppler ultrasound of blood flow velocity through the middle cerebral artery. While these approaches can inform on the general cerebrovascular tone, they do not deliver information on the dilatory properties of specific vessels across the cortex in the context of Aβ deposition. Indeed, it has been demonstrated that the dilation of the arterial vessels during hypercapnic conditions depends on the vessel’s location along the cerebrovascular tree.^32,33^ However, very little is known about the age and sex-specific interactions between vascular contractility along the cerebrovascular tree in the presence of CAA and tissue plaques during AD progression. The combined effects of sex hormones and Aβ deposition on the endothelial cells and smooth muscle, may result in sex-specific arterial dysfunction at different points along the vasculature. By measuring both global blood oxygenation and single-vessel dilations across the vascular tree during hypercapnic conditions, we can gain clinically relevant insights along with a greater understanding of the mechanisms driving global cerebrovascular changes reflected in the BOLD signal.

**Figure 1:**
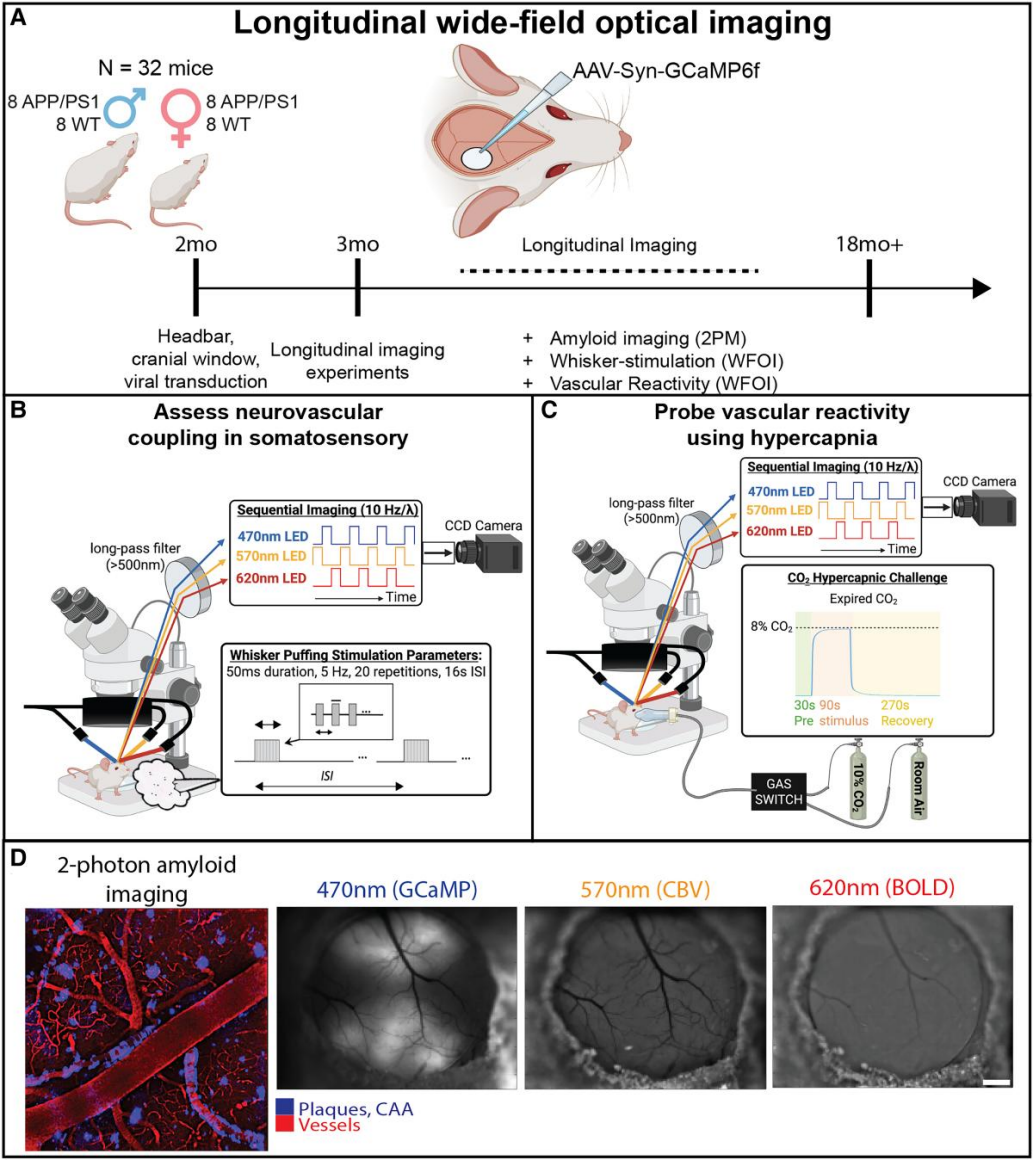
**Overview of the experimental approaches to investigate cerebrovascular function across the lifespan.** (**A**) APP/PS1 and control mice underwent a surgery to implant a head plate and cranial window for optical imaging, and GCaMP6f transduction into the right somatosensory, visual, and retrosplenial cortex. Monthly awake, widefield optical imaging was performed to assess neurovascular coupling and vascular reactivity. 2-photon microscopy was performed monthly on APP/PS1 mice to measure Aβ deposition. (**B**) Experimental setup for probing neurovascular coupling in the somatosensory cortex via whisker puffing stimulation. (**C**) Experimental setup for CO_2_ reactivity measured via hypercapnia challenge. (**D**) Example images from the two-photon (scale bar = 10 um) and the widefield microscopy (scale bar = 500 um). For the two-photon image, methoxy-04 was labelled for amyloid-β (Aβ), blood vessels were labelled with Sulforhodamine 101 (SR101). OIS-BOLD = deoxy-haemoglobin-weighted (blood oxygen level dependent) optical intrinsic signal; CBV = total haemoglobin-weighted (cerebral blood volume) optical intrinsic signal; AAV = adeno-associated virus; Syn = synapsin; GCaMP = GCaMP6f fluorescent signal; APP = Amyloid Precursor Protein; PS1 = mutant human presenilin 1; WT = Wild Type; LED = light emitting diode; CCD = charge-coupled device; ISI = inter-stimulus interval; CAA = cerebral amyloid angiopathy; WFOI = wide field optical imaging, 2PM = 2-photon microscopy. Parts of these figures were created with Biorender.com released under a Creative Commons Attribution-NonCommercial-NoDerivs 4.0 International license. https://app.biorender.com/profile/noah_schweitzer/templates/680a90c85d145dc1bda6c65b.

The present study aimed to examine age and sex-specific differences in neurovascular coupling and cerebrovascular reactivity for APP/PS1 and control mice across the near-lifespan (Fig. 1A). Neurovascular coupling was investigated via whisker stimulation, using GCaMP6f expression in barrel cortex neurons to measure neuronal activity, and haemoglobin-based optical intrinsic signal (OIS) to assess the evoked vascular response (Fig. 1B). To evaluate the vascular response independent from neuronal activity, we delivered 8% CO_2_ enhanced air mixture for hypercapnia stimulation (Fig. 1C). CO_2_ reactivity was measured using OIS, followed by the measurement of 3195 vessel diameters to assess single-vessel dilation in response to hypercapnia. Concomitantly, CAA vessel coverage and Aβ tissue plaque volume were longitudinally measured *in vivo* via two-photon microscopy (Fig. 1D) to link the observed vascular dysfunction to Aβ pathology in the AD mice. We hypothesized that we would observe innate sex differences in vascular function during healthy ageing of the control mice, and that those vascular sex differences will interact critically with CAA and tissue plaques to modify differentially the progression of brain dysfunction during the lifespan of the AD mice.

## Methods

### Animal preparation for optical imaging

All animal procedures were approved by the Division of Laboratory Animal Resources and Institutional Animal Care and Use Committee at the University of Pittsburgh and performed in accordance with the National Institutes of Health Guide for Care and Use of Laboratory Animals. The mouse strain used for this research project, B6C3-Tg(APPswe,PSEN1dE9)85Dbo/Mmjax, RRID:MMRRC_034829-JAX, was obtained from the Mutant Mouse Resource and Research Center (MMRRC) at The Jackson Laboratory, an NIH-funded strain repository, and was donated to the MMRRC by David Borchelt, Ph.D., McKnight Brain Institute, University of Florida.^34^ We used APP/PS1 transgenic mice, both male and female (Jackson Labs-MMRC) double transgenic for chimeric mouse/human amyloid precursor protein (Mo/HuAPP695swe) and mutant human presenilin 1 (PS1-dE9) protein directed to neurons.^34^ Control mice (B6C3, Jackson Labs) from the background of the AD mutations were age and sex matched (*N* = 8 Ad male, 8 Ad female, 8 WT male, 8 WT female for total of 32 mice across all groups). The APP/PS1 mouse model enables near-lifespan analysis of cerebrovascular function, as its gradual accumulation of Aβ in the brain parenchyma and vasculature more closely reflects chronic AD progression compared to other transgenic models.^35^ Sample size was determined based on prior studies using this model.^36^

Prior to data collection, each animal underwent a surgery at approximately 2 months of age to implant a head holder and a cranial window for optical imaging, as previously described.^37^ Briefly, mice were anaesthetized with ketamine/xylazine, dosed at 70 and 10 mg/kg, respectively. Body temperature was maintained at ∼37.0°C with thermal feedback. First, the fur from the scalp was shaved and the region was antiseptically cleaned with Betadine. Then, an approximately 1 × 1-cm area of skin was excised, and the bone exposed from the dorsal parietal skull. A 4 mm craniotomy over the right primary somatosensory cortex was performed and each animal was transduced with AAV-Syn-GCaMP6f (Penn Vector Core, Perelman School of Medicine, University of Pennsylvania) for expression of green fluorescence intracellular calcium reporter in all neurons in the retrosplenial, visual and somatosensory cortex (stereotaxic coordinates were determined by the Franklin and Paxinos mouse brain atlas^38^) at a depth of 150–300 µm. The injected viral volume was adjusted for viral titre with PBS to deliver the same number of viral particles per animal, resulting in 3–5 microliters per animal distributed over the three stereotactic cortical sites. The craniotomy was re-sealed with a glass coverslip, and an aluminium head-plate was fixed to the skull (Narishige, CP-2). Mice were given 4 weeks to recover post-surgery before imaging. Two weeks following surgery, mice were acclimated to head fixation on a treadmill for 1 h for 5 consecutive days to reduce rodent stress.

### Widefield optical imaging

#### Widefield optical image Acquisition

All whisker stimulation and hypercapnic experiments were performed in all animals under awake head-fixed conditions once per month. We utilized a mesoscopic-scale widefield microscope (MVX-10; Olympus, Tokyo, Japan, 2.5× magnification) for simultaneous imaging of neuronal Ca^2+^ transients and haemoglobin-based intrinsic optical signals. The illumination was conducted with three sequentially interleaved LED light sources driven by Arduino Uno microcontroller board communicating with a computer. Barrier filters were placed in front of each LED to restrict the spectral band to the desired wavelength: (1) blue for GCaMP6f excitation (470 ± 20 nm); (2) green for measuring total haemoglobin at an absorption isosbestic point (570 ± 7 nm); and (3) red for deoxy-haemoglobin-weighted imaging (620 ± 7 nm). Based on the molar extinction coefficients of oxyhaemoglobin and deoxyhaemoglobin, the OIS near 580 nm is an absorption isosbestic point and reflects the total haemoglobin comprising cerebral blood volume (CBV), herein referred as ‘OIS CBV’. The OIS measured with absorption at 620 nm is deoxyhaemoglobin-weighted and more sensitive to the blood oxygenation level, thus comparable to the BOLD fMRI signal and herein referred as ‘OIS BOLD’. Fluorescence and reflectance light from the brain was collected through a long-pass filter (>500 nm; ET500lp, Chroma Technology Corporation, VT, US) placed in the emission path before the digital cooled-CCD camera (CoolSnap HQ2; Photometrics, Princeton, NJ, USA) and images were sampled at 30 Hz (10 Hz per LED). The camera was controlled using MetaMorph software with an imaging matrix size of 260 **×** 348 pixels and field of view of 4.4 mm **×** 5.9 mm.

For whisker stimulation we used a flow-directed air puff delivered by Picospritzer (Parker-Hannifin Corporation) controlled by Master 9 (A.M.P.I.) pulse generator interfaced with computer and programmable through MATLAB software. The air puff pulse duration was 50 ms, the frequency was 5 Hz, and the stimulus length was 2 s. Mice were stimulated with a 40 s inter-stimulus interval (ISI) repeated 8 times or 16 s ISI repeated 20 times. For the hypercapnic experiment, we delivered a 10% CO_2_-enhanced air mixture and measured the end-tidal oxygen and CO2 concentration with a capnometer (Capnomac Ultima, Datex-Ohmeda Inc., Madison, WI) to ensure a desired 8% gas end-tidal concentration. The hypercapnia paradigm consisted of 30 s of baseline, 90 s of CO_2_ stimulation, and 270 s of recovery and repeated twice. During rest and recovery, the animal was administered medical-grade air.

#### Widefield optical image Analysis

All data were analysed using MATLAB (MathWorks, MA, USA). Camera images were first spatially binned by a factor of 2 (to 33.7 μm/pixel resolution), followed by motion and intensity correction. Motion correction consisted of a 2D Fourier-based sub-pixel, rigid registration using the first image from each experiment as reference. Intensity correction was performed on all images by regressing out the average intensity from regions placed over the skull bone or head bar from each pixel in the image to account for drifts and fluctuations in light source intensity.

For whisker stimulation, an activation map GCaMP6f signal change (ΔF/F_0_) was computed by the intensity difference between each pixel and the 2-s baseline period preceding each stimulus onset. A region of interest (ROI) in the whisker barrel region was extracted based on the area with the top 100 connected pixels with the highest activation. The peak fluorescence change was then calculated for GCaMP6f and OIS BOLD for this region.

For the hypercapnic challenge, a ROI was created by manually segmenting the entire brain coverage within the glass window. Then, large vessels were removed from the ROI by performing image segmentation based on K-means clustering and the resulted ROI that was corrected for low-frequency modulations to account for spatial inhomogeneity. After the large vessels were removed, tissue haemodynamic time-series for OIS BOLD and OIS CBV were calculated as the average signal for all pixels within the ROI at each frame. The vascular reactivity was then quantified as the peak change (ΔR/R_0_) of the OIS BOLD and OIS CBV signal during the stimulus. Separately, the vessel diameters were measured as the full-width-at-half-maximum (FWHM) of the vessel cross-sectional intensity profile. The per cent change in diameter was calculated as the peak absolute change during stimulus divided by the 30-s baseline period.

### 2-photon microscopy imaging

#### 2-photon microscopy image acquisition

One day before imaging, the mice were intraperitoneally injected with Methoxy-04 (1 mg/kg) to visualize Aβ deposition *in vivo*. Just before imaging, the mice were intraperitoneally injected with Sulforhodamine 101 (SR101) (Thermo Fisher Scientific, 0.2 μL g−1) to visualize the cerebral vasculature. Imaging was performed using an Ultima IV microscope coupled to an ultra-fast laser (Excite X3, Newport Spectra-Physics, Inc). Awake, head-fixed mice were placed on a custom-made frame treadmill with movable horizontal drums designed to accommodate mouse locomotion and minimize motion contamination of images. Images of the vasculature, CAA and Aβ tissue plaques were acquired using a 16X water immersion objective lens (0.80 NA, Nikon, Inc.) with the excitation laser tuned to 920 and 740 nm, respectively. For each imaging session, 2–4 images were acquired across the cortex with a maximum field-of-view of 1130 × 1130 μm (512 × 512 pixels), capturing depths of up to 400 μm (layers 1–4 of the cortex). This corresponds to an in-plane resolution of 2.207 μm/pixel with a step size of 3 μm between image stacks.

#### CAA and plaque volume quantification

All data were analysed using MATLAB (MathWorks, MA, USA). The two-photon z-stacks were first pre-processed by performing median filtering (3-by-3 neighbourhood), gaussian smoothing (σ = 0.4), and removing the low-frequency intensity fluctuations extracted from the average image. Finally, volume intensity correction was performed in the z-direction where each quantile of the volume was normalized to the signal intensity of the first quantile closest to the cortical surface. For CAA quantification, arteries and arterioles with Aβ deposition were identified from the overlap with the rhodamine vascular channel before the calculation of maximum intensity projection (MIP). Vessels with CAA were manually segmented from the MIP, and pixels categorized as CAA were determined by thresholding 1.5 standard deviations above the mean intensity of the Methoxy X04 within the vessel. The percentage vessel coverage of CAA with respect to the total vessel area in the MIP was determined by thresholding. Aβ tissue plaque quantification started with the removal of CAA pixels from the z-stack volume followed by segmentation of plaques based on a threshold of greater than 0.6 times the maximum intensity. Tissue plaques were identified as segmented pixels with connected components greater than 15 pixels, and the total volume of tissue plaques was calculated from the segmented pixels. Thresholds of 1.5 standard deviations above the mean intensity for CAA images and 0.6 times the maximum intensity in tissue plaque volumes were selected to closely reflect morphology of CAA and tissue plaques across sample images, respectively. These thresholds enabled a robust, semi-automated method that was consistent across images and minimized selection bias. Measures of CAA vessel coverage and tissue plaque volume were averaged over the imaging session to compute a global average for each time point in each mouse.

### Statistical analysis

All statistical analyses were performed using R (version 4.3.1 https://www.R-project.org). Linear mixed effects regression models were used to assess the effects of age, sex, genotype (APP/PS1 versus control), and their interactions on measured outcomes across all experiments and controlling for each mouse as a random effect. For single-vessel dilations, the Johnson-Neyman test was utilized to examine the conditional effect of age on dilatory response versus baseline diameter for AD females and AD males, respectively.^39^ A medial split was performed on arterial vessel baseline diameters to classify vessels as small or large, and then one way *t-*tests were performed between sex and age (binned by 11 months of age). All mice underwent longitudinal imaging, yet not every animal contributed data at every time point due to occasional dropped imaging sessions or attrition over time (e.g. bone regrowth, inflammation, or compromised cranial windows). Images were only excluded if excessive motion was present. In particular, excessive motion in young mice during whisker puff and hypercapnia-induced anxiety motion in old mice. Supplementary Fig. 1 summarizes the cumulative mouse attrition per group, as well as the amount of excluded datapoints per genotype/sex. On average, mice were imaged for at 5.4 ± 3.1 time points, with a mean interval 1.52 ± 0.5 months between imaging sessions. All analyses were performed in a blinded manner.

## Results

### Neurovascular coupling response to whisker stimulus is diminished in AD mice

**Figure 2:**
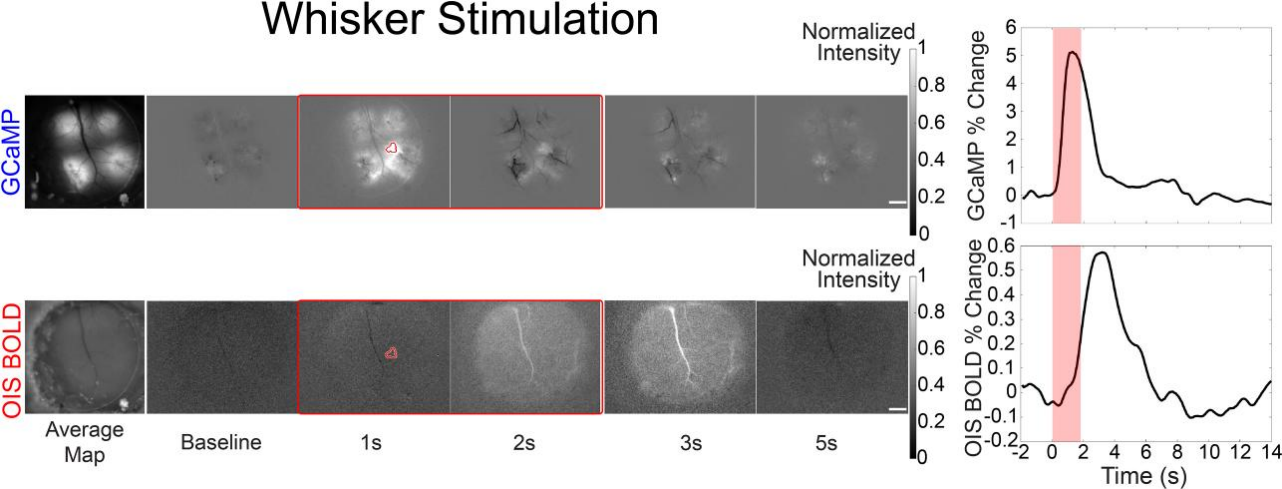
**Example of the neurovascular coupling during whisker puff measured by GCaMP and OIS BOLD changes in the barrel cortex.** The corresponding time series of fluorescent and OIS change presented in the right column were extracted from the activated whisker barrel (shaded region of interest). Scale bar = 500 um. OIS-BOLD = deoxy-haemoglobin-weighted (blood oxygen level dependent) optical intrinsic signal; GCaMP = GCaMP6f fluorescent signal.

**Figure 3:**
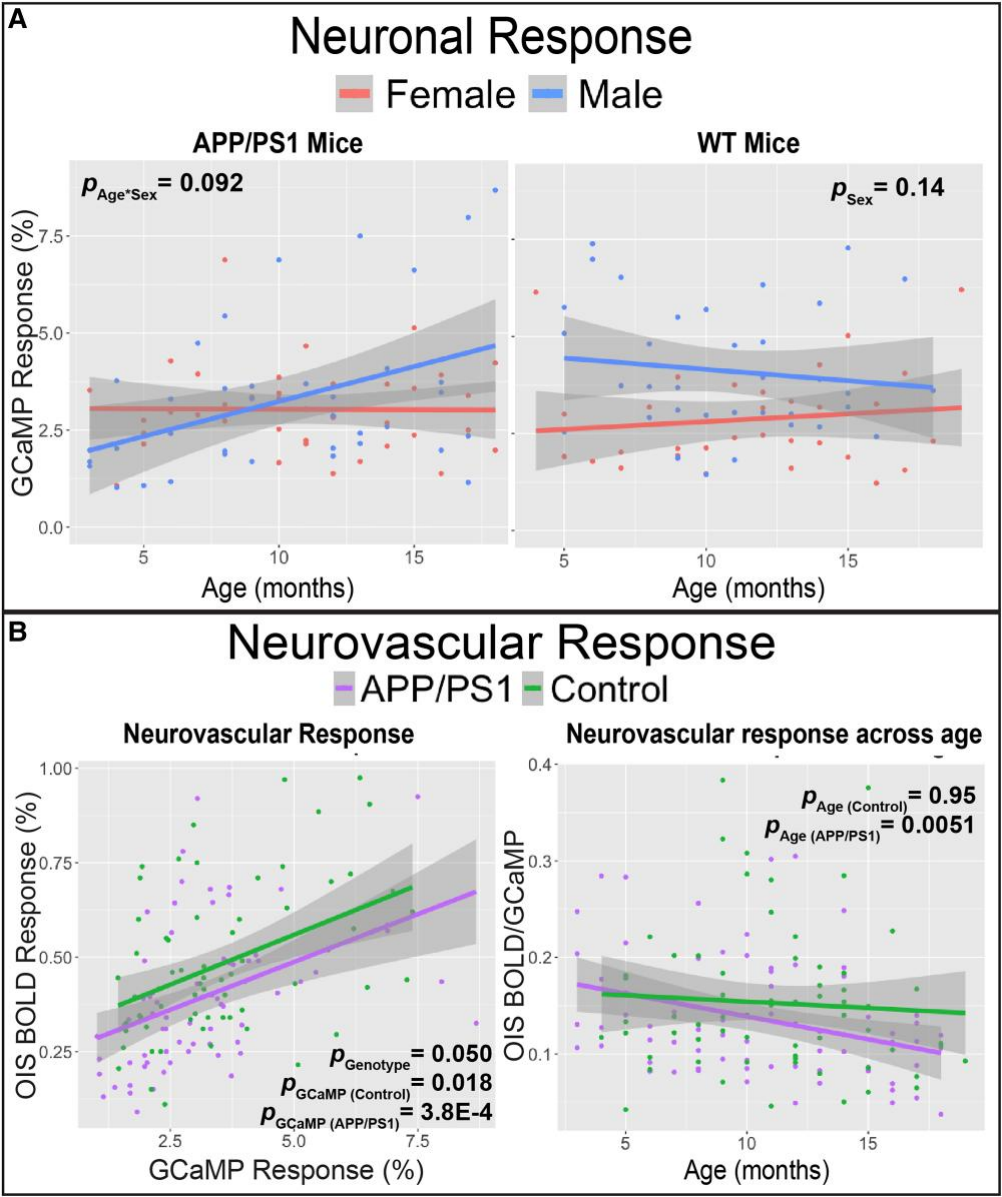
**The neurovascular coupling response is attenuated with age in AD mice.** (**A**) GCaMP neuronal response has a trend to increase with age for AD males but not AD females (left panel, *N* = 77 imaging sessions from 16 mice); Age*Sex interaction effect β = 0.14, 95% β confidence interval (−0.023, 0.30), ANOVA F(1, 73) = 2.91, *P* = 0.092. No sex difference in GCaMP response across age is observed for control mice (right panel, *N* = 68 imaging sessions from 16 mice); Sex main effect β = 1.16, 95% β confidence interval (−0.20, 2.53), ANOVA F(1, 65) = 2.89, *P* = 0.14. Multivariate linear mixed effect regression was used to test the interaction between age and sex, and the main effect of sex while controlling for age and a random effect for each mouse (Supplementary Table 1). (**B**) The haemoglobin-based OIS BOLD response with respect to the GCaMP activation is lower for AD mice compared to control mice (left; Genotype main effect β = 0.077, 95% β confidence interval (0.0039, 0.15), ANOVA F(1, 141) = 4.34, *P* = 0.050) and the ratio of OIS BOLD:GCaMP is attenuated with age for AD but not control mice (right; Age_AD mice_ main effect β = −0.0047, 95% β confidence interval (−0.0080, −0.0015), ANOVA F(1, 73) = 8.38, *P* = 0.0051; Age_WT mice_ main effect β = −0.00018, 95% β confidence interval (−0.0052, 0.0049), ANOVA F(1, 65) = 0.0048; *P* = 0.95). Multivariate linear mixed effect regression was used to assess the effect of GCaMP response on OIS BOLD response within each genotype while controlling for sex and a random effect for each mouse (left panel, *N* = 145 imaging sessions from 32 mice; Supplementary Table 3), and the effect of age on the OIS BOLD:GCaMP ratio while controlling for sex and a random effect for each mouse (right panel, *N* = 145 imaging sessions from 32 mice; Supplementary Table 4). An imaging session is one awake, head-fixed acquisition for a single mouse at a single longitudinal time point with an intact cranial window; whisker-evoked blocks are collected and session-level GCaMP and OIS-BOLD peaks are the mean of trial-wise maxima within the barrel-cortex ROI (top-100 connected pixels). In all panels, each data point represents a quantified measurement from a single imaging session for one mouse. Shaded area represents 95% confident interval. OIS-BOLD = deoxy-haemoglobin-weighted (blood oxygen level dependent) optical intrinsic signal; GCaMP = GCaMP6f fluorescent signal; APP = Amyloid Precursor Protein; PS1 = mutant human presenilin 1; WT = Wild Type.

We first examined the lifespan trajectories of the neurovascular coupling components in the somatosensory barrel cortex of AD and control mice (Fig. 2; Supplementary Fig. 2). Neuronal excitation was defined as the peak GCaMP response to whisker stimulation. To assess sex differences, we tested the effect of sex on peak neuronal excitation while controlling for age within each genotype (Supplementary Table 1—Model 1). No sex differences were observed for neuronal excitation across age for control mice (*P* = 0.14, Fig. 3A) or AD mice (*P* = 0.97). We next tested the interaction between age and sex on peak neuronal excitation within each genotype (Supplementary Table 1- Model 2). We observed a trend for AD mice where the neuronal excitation increased with age for the AD male mice, but not for the AD females (Sex × Age interaction *P* = 0.092; Fig. 3A).

Next, we examined whether there were sex differences in AD pathology across age. We tested the effect of sex on CAA vessel coverage and Aβ plaque volume while controlling for age. We observed that AD males exhibited slightly higher plaque volume compared to AD females (*P* = 0.049), whereas sex differences in CAA were marginally non-significant (*P* = 0.052; Supplementary Fig. 3A). To further investigate how AD pathology relates to neuronal activity, we assessed the effects of CAA vessel coverage and Aβ plaque volume on peak neuronal excitation (Supplementary Table 2—Models 1 and 2), as well as potential sex interactions (Models 3 and 4). No significant associations were observed between Aβ plaque volume and neuronal excitation in either AD males (*P* = 0.34) or AD females (*P* = 0.49). A sex × CAA vessel coverage interaction trend was observed in AD mice (*P* = 0.076), such that increased CAA burden was associated with greater neuronal excitation in AD males but not in AD females (Supplementary Fig. 3B).

We then probed the vascular component of the neurovascular response, measured as the peak OIS BOLD response with respect to the peak GCaMP response. To assess the contributions of the neuronal activity and sex, we tested the effects of GCaMP response and sex on OIS BOLD within each genotype (Supplementary Table 3—Model 1). In both AD and control mice, peak OIS BOLD was significantly associated with GCaMP response (*P* = 3.8E-4 and 0.018 for AD and WT, respectively; Fig. 3B), with no sex differences observed for either genotype (Supplementary Fig. 4A). To test the effect of genotype on OIS BOLD, we used a separate model controlling for sex and GCaMP response (Supplementary Table 3- Model 2). In this analysis, AD mice exhibited significantly lower OIS BOLD responses compared to control mice (*P* = 0.050; Fig. 3B).

We then probed the neurovascular response across age by examining the ratio of the BOLD:GCaMP response. We tested the effects of age and sex on BOLD:GCaMP ratio within each genotype (Supplementary Table 4- Model 1). The BOLD:GCaMP ratio decreased with age for AD mice (*P* = 0.0051) but not control mice (*P* = 0.95; Fig. 3B). We observed no sex differences for the BOLD:GCaMP ratio across age for AD mice (*P* = 0.71) nor WT mice (*P* = 0.85; Supplementary Fig. 4B). Finally, we examined the association between AD pathology on the BOLD:GCaMP ratio while controlling for sex (Supplementary Table 4—Models 2 and 3). The BOLD:GCaMP ratio was not significantly associated with Aβ plaque volume (*P* = 0.84) or CAA vessel coverage (*P* = 0.23; Supplementary Fig. 3C). Individual longitudinal trajectories of the neurovascular response to whisker puffing are displayed on Supplementary Fig. 6A.

### CO_2_ reactivity is diminished in AD mice

**Figure 4:**
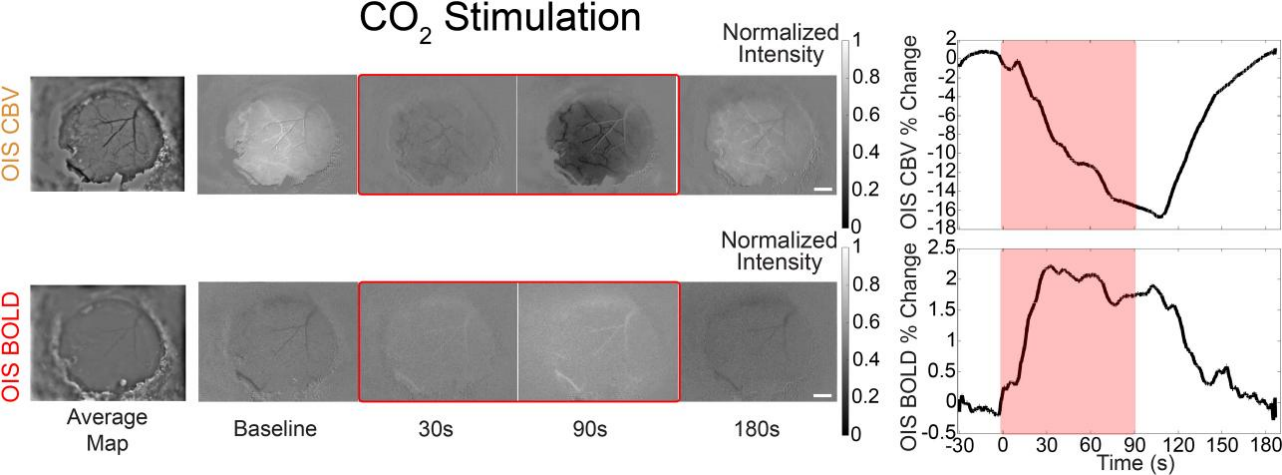
**Example of the vascular reactivity response to hypercapnia challenge measured by OIS BOLD and OIS CBV peak fluorescent change**. Scale bar = 500 um. OIS-BOLD = deoxy-haemoglobin-weighted (blood oxygen level dependent) optical intrinsic signal; CBV = total haemoglobin-weighted (cerebral blood volume) optical intrinsic signal.

**Figure 5:**
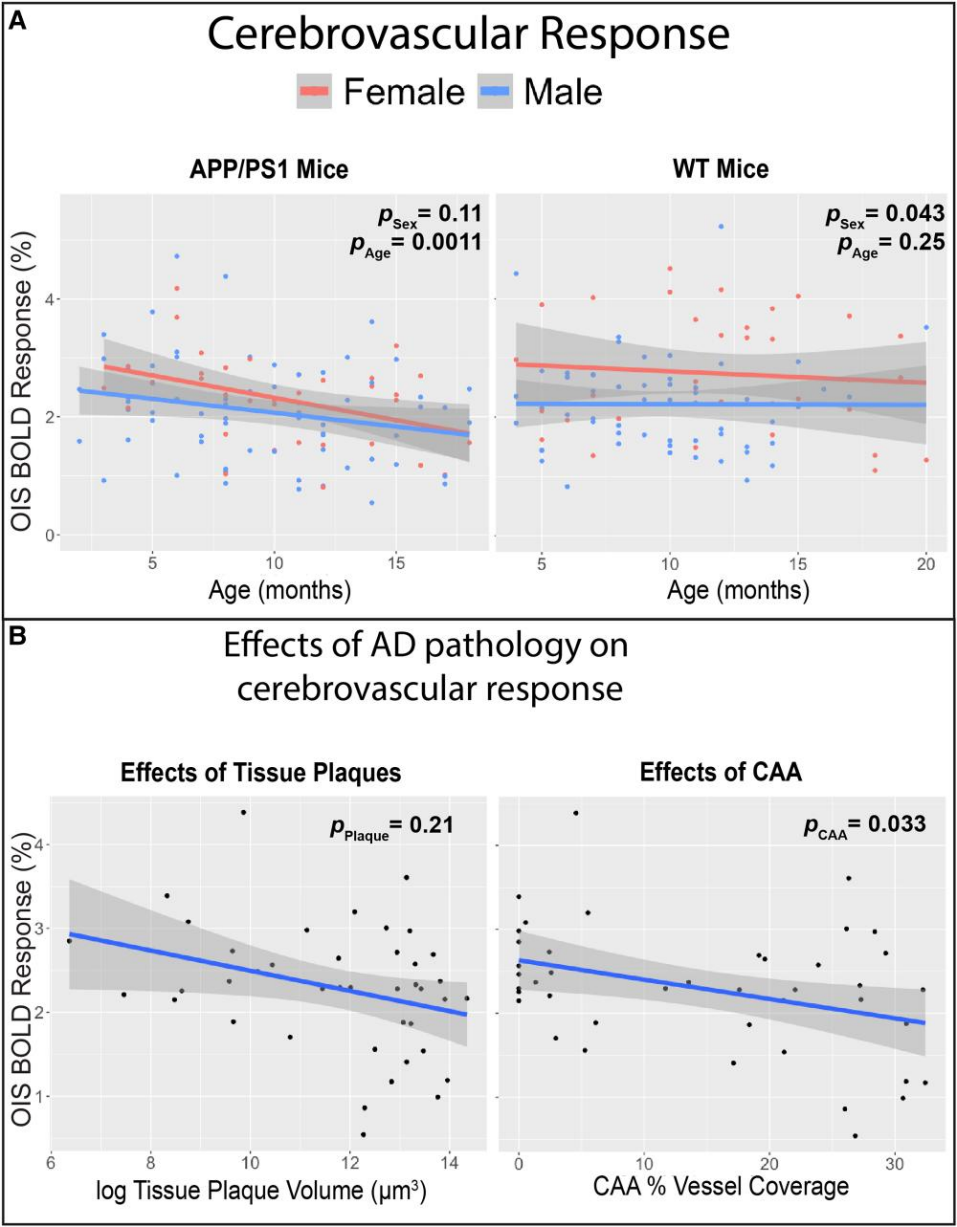
**CO_2_ reactivity is attenuated with age in AD mice.** (**A**) OIS BOLD response significantly decreases with age in AD mice (Age main effect β = −0.067, 95% β confidence interval (−0.11, −0.028), ANOVA F(1, 102) = 11.5, *P* = 0.0011) but not control mice (Age main effect β = −0.024, 95% β confidence interval (−0.064, 0.017), ANOVA F(1, 89) = 1.35, *P* = 0.25). Control females have higher OIS BOLD response compared to control males and AD females, regardless of age (Sex main effect β = −0.65, 95% β confidence interval (−1.24, −0.057), ANOVA F(1, 89) = 4.75, *P* = 0.043). Multivariate linear mixed effect regression was used to test the age and sex effect on OIS BOLD within AD mice (left panel, *N* = 108 imaging sessions from 16 mice) and WT mice (right panel, *N* = 97 imaging sessions from 16 mice; Supplementary Table 5), controlling for a random effect for each mouse. (**B**) The attenuated vascular reactivity in AD mice is associated with CAA vessel coverage (CAA main effect β = −0.026, 95% β confidence interval (−0.050, −0.0024), ANOVA F(1, 39) = 5.00, *P* = 0.033) but not tissue plaque volume (tissue plaque main effect β = −0.082, 95% β confidence interval (−0.21, −0.049), ANOVA F(1, 39) = 1.63, *P* = 0.21). Multivariate linear mixed effect regression was used to test the effect of CAA (left panel) and log tissue plaque volume (right panel) on OIS BOLD within AD mice (*N* = 42 imaging sessions from 16 mice; Supplementary Table 6) while controlling for sex and a random effect for each mouse. An imaging session is one awake, head-fixed acquisition for a single mouse at a single longitudinal time point with an intact cranial window; the hypercapnia paradigm is 30-s baseline followed by a 90-s 10% CO_2_ and then a 270-s recovery repeated twice, large surface vessels are masked from the tissue ROI, and session-level OIS-BOLD peaks are the mean of the two epoch peaks, with each dot representing one session. In all panels, each data point represents a quantified measurement from a single imaging session for one mouse. Shaded area represents 95% confident interval. OIS-BOLD = deoxy-haemoglobin-weighted (blood oxygen level dependent) optical intrinsic signal; CBV = total haemoglobin-weighted (cerebral blood volume) optical intrinsic signal; CAA = Cerebral Amyloid Angiopathy; APP = Amyloid Precursor Protein; PS1 = mutant human presenilin 1; WT = Wild Type; AD = Alzheimer’s Disease.

To probe the vascular response independent from neuronal activity, we delivered a 10% CO_2_ in air mixture with an 8% end-tidal CO_2_ concentration to induce hypercapnia (Fig. 4). CO_2_ reactivity was assessed by measuring the peak change in OIS BOLD and OIS CBV responses. We first tested the effects of age and sex on OIS BOLD and CBV using linear regression (Supplementary Table 5-Model 1). Control females had a significantly higher OIS BOLD response across age compared to males (*P* = 0.043), and these innate sex differences were lost in AD mice (*P* = 0.11; Fig. 5A). No significant sex differences were observed in OIS CBV responses for either AD (*P* = 0.39) or control mice (*P* = 0.49; Supplementary Fig. 5A). Age-related declines in CO_2_ reactivity were evident in AD mice, with both OIS BOLD (*P* = 0.0011) and OIS CBV (*P* = 0.015) responses significantly diminished with age. In contrast, no significant age-related changes were observed in control mice for either BOLD (*P* = 0.25) or CBV (*P* = 0.078) responses (Fig. 5A; Supplementary Fig. 5A). Furthermore, we detected significant age × genotype interaction effects for both OIS BOLD (*P* = 0.036) and CBV responses (*P* = 0.0029), indicating that CO_2_ reactivity declined more rapidly with age in AD mice compared to controls (Supplementary Table 5—Model 2). Individual longitudinal trajectories of the cerebrovascular response to CO_2_ challenge are displayed on Supplementary Fig. 6B.

We next tested the effects of AD pathology on the OIS BOLD and CBV responses through linear regression analyses controlling for sex (Supplementary Table 6). The OIS BOLD response was significantly associated with CAA vessel coverage (*P* = 0.033) but not with Aβ plaque volume (*P* = 0.21; Fig. 5B). The OIS CBV response trended towards significance with CAA vessel coverage (*P*  *=* 0.056) but not with Aβ plaque volume (*P*  *=* 0.69; Supplementary Fig. 3B).

### AD and sex-specific differences in CO_2_ reactivity diameter changes

**Figure 6:**
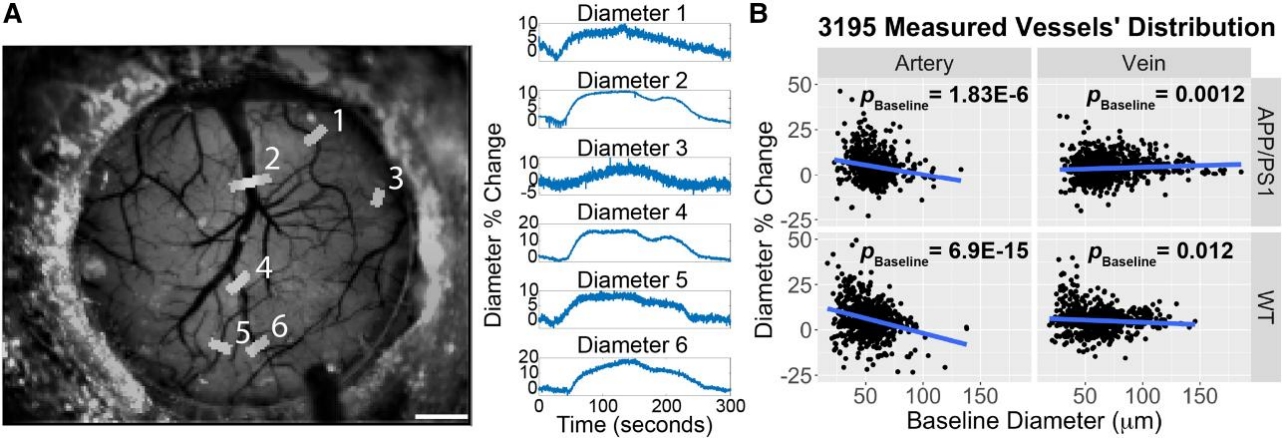
**Example vessel diameter measurements during hypercapnia challenge.** (**A**). Scale bar = 500 um. (**B**) Small arterial vessels experience a higher peak dilation compared to larger arterial vessels; baseline diameter main effect β = −0.15, 95% β confidence interval (−0.18, −0.12), ANOVA F(1, 1377) = 85.4, *P* = 2.2E-17. Multivariate linear mixed effect regression was used to test the effect of baseline diameter on diameter % change (*N* = 3195 vessel measurements from 32 mice; 1386 arterial and 1809 venous vessels) while controlling for sex and age with a random effect for each mouse. An imaging session is one awake, head-fixed acquisition for a single mouse at a single longitudinal time point with an intact cranial window; during the session’s hypercapnia block all resolvable arterioles and venules are measured for baseline diameter and peak per cent change during CO_2_ challenge. In all panels, each data point represents a single vessel peak dilation from a single imaging session. Shaded area represents 95% confident interval. APP = Amyloid Precursor Protein; PS1 = mutant human presenilin 1; Wild Type = WT.

To further probe how AD is associated with CO_2_ reactivity changes, we measured the diameters of 3195 vessels (1386 arterial and 1809 venous from 32 mice) during the hypercapnic experiment (Fig. 6A). We observed a significant negative association between baseline diameter and % change in vessel diameter (*P* < 0.001) for arterial vessels across all groups, indicating that the smaller arterial vessels exhibited greater dilation (Fig. 6B). Additionally, we observed a positive association between baseline diameter and % change in vessel diameter in venous vessels for APP/PS1 mice (*P* = 0.0012) and a negative association for WT mice (*P* = 0.012; Fig. 6B). We first tested the effects of age and sex on baseline vessel diameter within each genotype (Supplementary Table 7-Model 1). No significant associations were observed between age and arterial baseline diameter in AD (*P* = 0.43) or WT mice (*P* = 0.064). No sex differences were observed for baseline arterial diameter for both WT mice (*P* = 0.24) and AD mice (*P* = 0.68). In venous vessels, baseline diameter increased significantly with age in AD mice (*P* = 0.033) but not WT mice (*P* = 0.075). No sex differences were observed for venous baseline diameter in WT mice (*P* = 0.16), or AD mice (*P* = 0.29).

**Figure 7:**
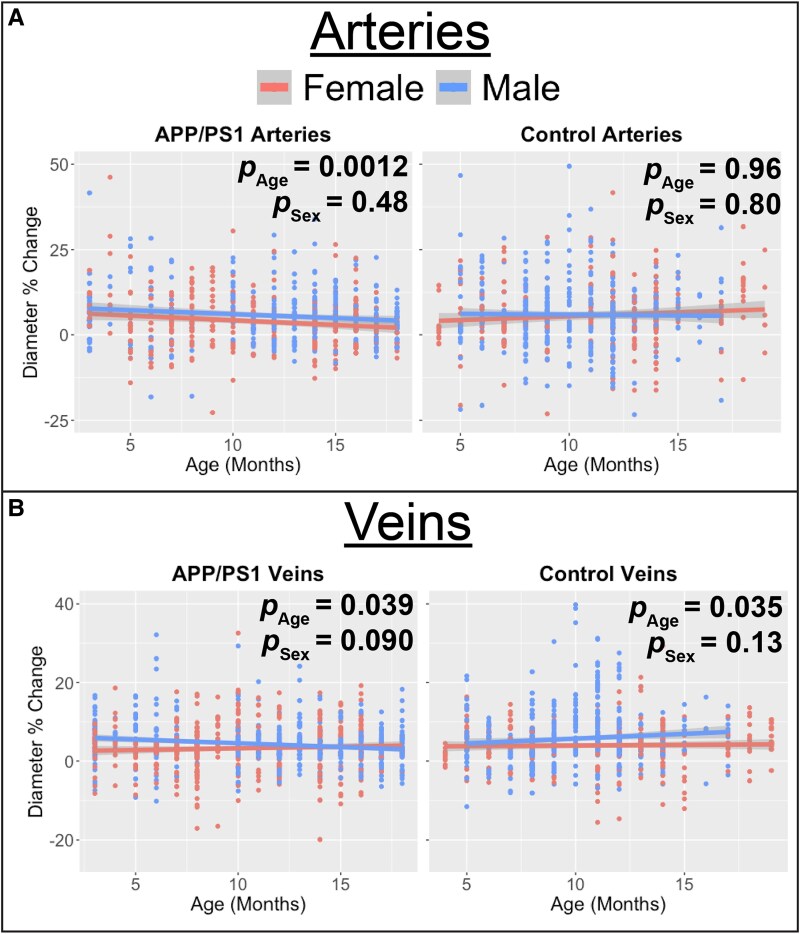
**3195 vessel diameter measurements during hypercapnia challenge across age reveal significant decrease in arterial dilations for AD mice.** (**A**) Arterial dilation decreases with age for AD mice (age main effect β = −0.29, 95% β confidence interval (−0.46, −0.12), ANOVA F(1, 689) = 10.82, *P* = 0.0012) but not control mice (age main effect β = −0.0057, 95% β confidence interval (−0.25, 0.23), ANOVA F(1, 685) = 0.0022, *P* = 0.96). Multivariate linear mixed effect regression was used to test the effect of sex and age on arterial vessel diameter % change in AD mice (left panel, *N* = 697 vessel measurements from 16 mice) and WT mice (right panel, *N* = 689 vessel measurements from 16 mice) while controlling for baseline diameter and a random effect for each mouse (Supplementary Table 8). (**B**) Venous dilation decreases with age in AD mice and increases with age in WT mice (Age_AD mice_ main effect β = −0.12, 95% β confidence interval (−0.22, −0.0062), ANOVA F(1, 1027) = 4.30, *P* = 0.038; Age_WT mice_ main effect β = 0.17, 95% β confidence interval (0.012, 0.32), ANOVA F(1, 774) = 4.5, *P* = 0.035). Multivariate linear mixed effect regression was used to test the effect of sex and age on venous vessel diameter % change in AD mice (left panel, *N* = 1031 vessel measurements from 16 mice) and WT mice (right panel, *N* = 778 vessel measurements from 16 mice) while controlling for baseline diameter amd a random effect for each mouse (Supplementary Table 8). An imaging session is one awake, head-fixed acquisition for a single mouse at a single longitudinal time point with an intact cranial window; during the session’s hypercapnia block all resolvable arterioles and venules are measured for baseline diameter and peak per cent change during CO_2_ challenge. In all panels, each data point represents a single vessel peak dilation from a single imaging session. Shaded area represents 95% confident interval. APP = Amyloid Precursor Protein; PS1 = mutant human presenilin 1; Wild Type = WT.

We then tested the effects of age and sex on % change in arterial and venous vessel diameter within each genotype, controlling for baseline diameter (Supplementary Table 8- Model 1). AD mice exhibited a significant decrease in arterial vessel diameter % change with age (*P* = 0.0012), whereas no such effect was observed in control mice (*P* = 0.96 Fig. 7A). We observed no sex differences for AD mice (*P* = 0.48) or control mice (*P* = 0.80). For venous vessels, dilation significantly decreased with age in AD mice (*P* = 0.039) and control mice (*P* = 0.035; Fig. 7B). No sex differences were observed for AD mice (*P* = 0.090) or in WT mice (*P* = 0.13).

**Figure 8:**
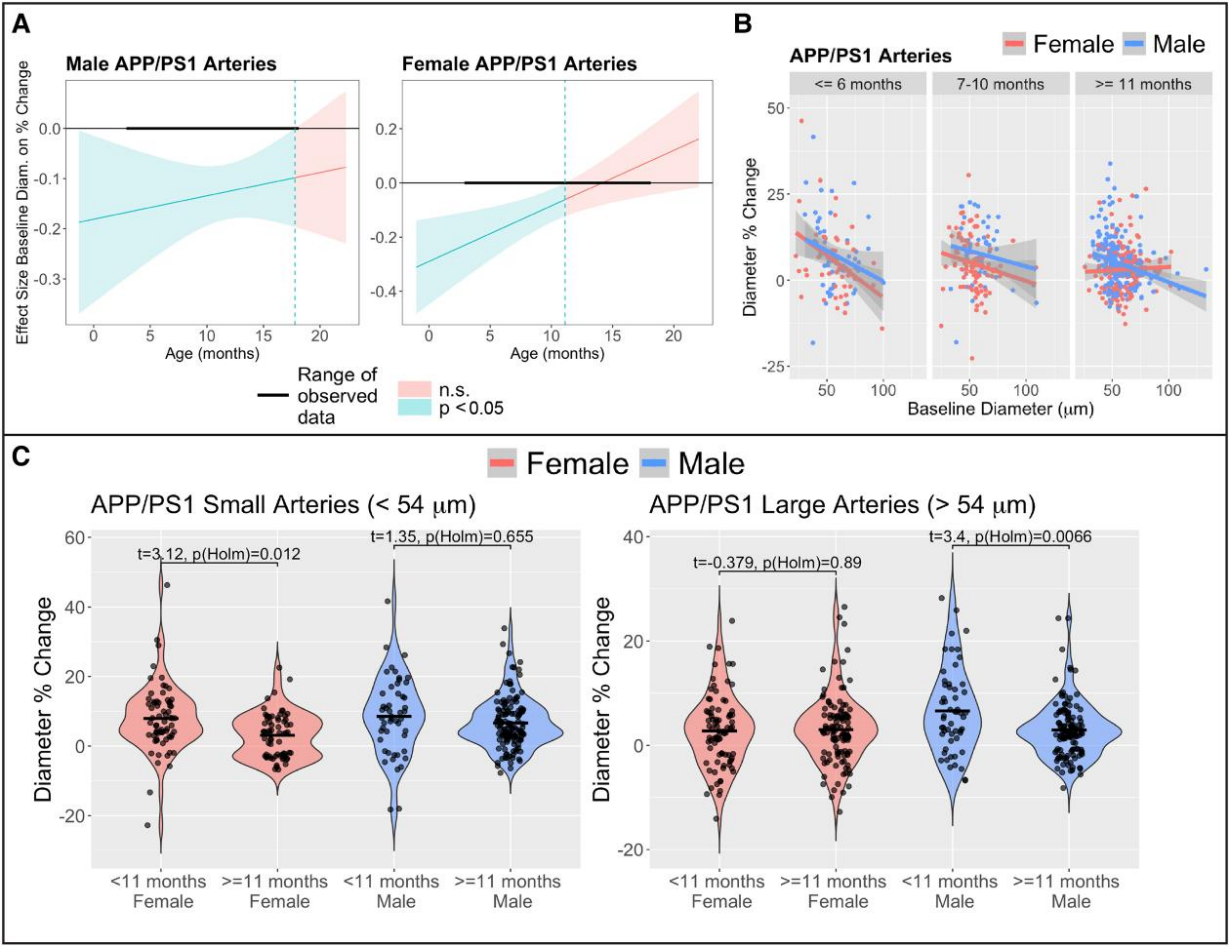
**AD males experienced diminished dilation in large arteries, while AD females showed greater impairment in small arteries.** (**A**) The Johnson-Neyman test was utilized to probe and visualize the conditional effect of age on dilatory response versus baseline diameter. AD females, but not AD males, have a significant attenuation in the association between peak arterial dilation and baseline diameter, with the association becoming insignificant around 11 months of age. Multivariate linear regression was used for the Johnson-Neyman test, assessing the interaction effect between baseline arterial diameter and age on diameter % change in AD males (left panel, *N* = 334 vessel measurements from 8 mice) and AD females (right panel, *N* = 363 vessel measurements from 8 mice). (**B**) A visualization of the association between peak arterial dilation and baseline diameter binned by age points towards small arterial dilation diminishing with age. Basic linear regression was used to visualize trend lines at each time point. (**C**) A medial split on arterial diameter (54 μm) was performed to classify vessels as small or large. Small arterial dilation significantly decreases with age for AD females but not AD males, whereas large arterial dilation significantly decreases for AD males but not AD females. Group differences in diameter per cent change were assessed using linear mixed effect models with mouse as a random effect and each sex-by-age group as a fixed effect, following by Holm-adjusted pairwise contrasts. An imaging session is one awake, head-fixed acquisition for a single mouse at a single longitudinal time point with an intact cranial window; during the session’s hypercapnia block all resolvable arterioles and venules are measured for baseline diameter and peak per cent change during CO_2_ challenge. In all panels, each data point represents a single vessel peak dilation from a single imaging session. Shaded area represents 95% confident interval. APP = Amyloid Precursor Protein; PS1 = mutant human presenilin 1.

We then investigated which arterial vessels’ response diminished with age. We tested the interaction effect between baseline diameter and age on arterial vessel dilatory response within AD males and females. For AD females, we observed that the association between baseline diameter and its corresponding dilatory response diminished with age (Age × baseline diameter interaction effect *P* = 0.0048) but this interaction was not significant for AD males (*P* = 0.50). The Johnson-Neyman test was utilized to examine the conditional effect of age on dilatory response versus baseline diameter for AD females. We observed that the effect size between baseline diameter on the dilatory response lost significance (*P* < 0.05) around 11 months of age for AD females (Fig. 8A). When plotting the association between baseline diameter and its dilatory response binned by age, we observed that the smallest arterial vessels had a diminished dilatory response for AD females at ≥ 11 months of age (Fig. 8A and B). To probe this association further, we performed a medial split on arterial vessel baseline diameter (median arterial diameter = 53.7 μm) to classify arterial vessels as small or large. For the small arterial vessels, AD females experienced a decreased dilatory response after 11 months of age compared to younger than 11 months (*P* = 0.012) whereas there was no significant change in the dilatory response for AD males (*P* = 0.66; Fig. 8C). For the large arterial vessels, AD males experienced a decreased dilatory response after 11 months of age compared to younger than 11 months (*P* = 0.0066) whereas there was no significant change in the dilatory response for AD females (*P* = 0.89; Fig. 8C).

**Figure 9:**
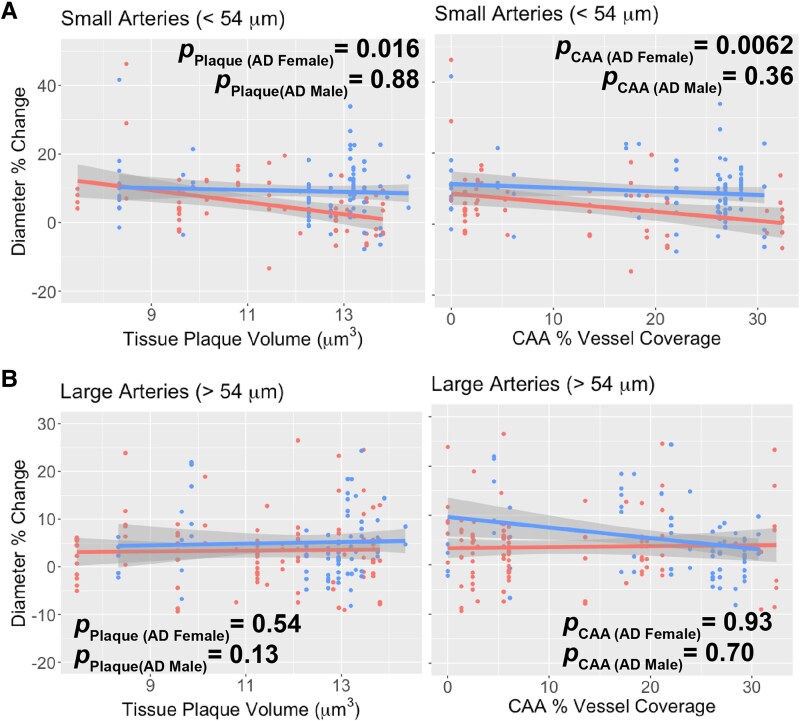
**Sex-specific arterial dysfunction is associated with AD pathology.** (**A**) Decreased small arterial dilation in AD females was associated with both CAA vessel coverage (left; CAA main effect β = −0.33, 95% β confidence interval (−0.56, −0.097), ANOVA F(1, 60) = 8.06, *P* = 0.0062) and tissue plaque volume (right; tissue plaque main effect β = −1.31, 95% β confidence interval (−2.36, −0.26), ANOVA F(1, 57) = 6.21, *P* = 0.016). (**B**) Decreased large arterial dilation in AD males was not associated with CAA vessel coverage (left; CAA main effect β = −0.065, 95% β confidence interval (−0.39, 0.26), ANOVA F(1, 79) = 0.16, *P* = 0.70) or tissue plaque volume (right; tissue plaque main effect β = 2.07, 95% β confidenEce interval (−0.29, 4.44), ANOVA F(1, 79) = 3.04, *P* = 0.13). In (A, B), multivariate linear mixed effect regression was used to test the effect of arterial diameter % change on CAA (*N* = 161 vessel measurements from 8 mice) and tissue plaques (*N* = 158 vessel measurements from 8 mice) in both sexes (Supplementary Table 9) controlling for mouse as a random effect. An imaging session is one awake, head-fixed acquisition for a single mouse at a single longitudinal time point with an intact cranial window; during the session’s hypercapnia block all resolvable arterioles and venules are measured for baseline diameter and peak per cent change during CO_2_ challenge. In all panels, each data point represents a single vessel peak dilation from a single imaging session. Shaded area represents 95% confident interval. CAA = Cerebral Amyloid Angiopathy; APP = Amyloid Precursor Protein; PS1 = mutant human presenilin 1; WT = Wild Type.

Finally, we tested the effect of CAA vessel coverage (Supplementary Table 9- Model 1) and Aβ plaque volume (Supplementary Table 9- Model 2) on small and large arterial vessel dilation while controlling for baseline diameter within each sex. For small arterial vessels, increased Aβ plaque volume (*P* = 0.016) and CAA vessel coverage (*P* = 0.0062) were associated with decreased dilatory response in small arterial vessels for AD females but not for AD males (CAA, *P* = 0.36; Tissue plaques, *P* = 0.88; Fig. 9A). Neither CAA vessel coverage or Aβ plaque volume was associated with a decreased dilatory response in large arterial vessels for AD males (CAA, *P* = 0.70; Tissue plaques, *P* = 0.13) or AD females (CAA, *P* = 0.93; Tissue plaques, *P* = 0.54; Fig. 9B).

## Discussion

In this near-lifespan longitudinal study, we investigated age and sex-specific differences in neurovascular coupling in the somatosensory cortex as well as global CO_2_ reactivity for APP/PS1 transgenic mice and controls. When examining neurovascular coupling during whisker puffing, we found that the same level of neuronal activation evoked lower haemodynamic response in AD mice compared to controls. This diminished haemodynamic response was decreasing further with old age in both AD males and females. We also observed a trend that the evoked neuronal activity increased with age only in AD male mice, and no sex differences were observed in the neurovascular response. In measures of CO_2_ reactivity during a hypercapnia challenge, we observed a diminished response with age in AD mice, but not in controls. Additionally, control females exhibited higher vascular reactivity compared to control males, regardless of age but this innate higher reactivity was lost in the AD females. We then probed how sex and AD impacted vessel-specific dilatory responses during hypercapnia challenge by measuring 3195 vessel diameters across age. We found that while both AD males and females showed a reduced dilatory response with age compared to controls, AD females exhibited this decrease in small arterial vessels (<54 μm diameter), whereas AD males experienced a diminished dilatory response in larger arterial vessels (>54 μm diameter). Diminished global CO_2_ reactivity and dilatory responses were associated with CAA but not Aβ tissue plaque volume, emphasizing the importance of considering CAA when examining vascular dysfunction in AD. Our findings identify sex-specific pathophysiology of vascular dysfunctions in the presence of Aβ deposits, with important implications for developing biomarkers and therapies targeting Aβ-related dysfunction in AD.

By leveraging both neurovascular coupling and the independent vascular response to hypercapnia, our study provides a more detailed understanding of the distinct, sex-specific effects on neurons and vascular cells. Neurovascular coupling and CO_2_-induced reactivity rely on distinct signalling pathways and their separate quantification across the lifespan can provide clues for the mechanistic origins of vascular contributions to AD.^9^ Neurovascular coupling is driven by a sequence of highly coordinated multicellular events in which neurons and astrocytes release various vasoactive mediators (e.g. prostanoids, neuropeptides, neurotransmitters, nitric oxide) that trigger both localized microvascular and upstream arterial responses to support active sites.^8^ In contrast, hypercapnic vasodilation is thought to operate independently of neuronal control, resulting from acidification and elevated arterial partial pressure of CO_2_ that lead to endothelial cell signalling and vascular smooth muscle cells (vSMC) relaxation.^40^ This process is driven directly by CO_2_ and pH changes, that initiate nitric oxide and hydrogen sulphate pathways and mediate the vasodilation of precapillary arterioles.^8,41^ We observed no sex differences in the neurovascular coupling response relative to neuronal activity in control mice. In contrast, hypercapnia elicited a greater cerebrovascular response in control females than males, a difference diminished in AD. Together, our differential findings suggest that the pathways of Aβ-driven vascular dysfunction may vary between sexes, with neuronally-controlled vascular responses more diminished in AD males, while CO_2_ reactivity is more affected in AD females.

Increasing evidence has linked vascular dysfunction early in the disease progression of AD.^2,3^ We observed a decline in neurovascular coupling in response to sensory stimuli, consistent with prior studies in both mice^42-45^ and humans with heightened AD pathology.^46,47^ Additionally, we detected significant attenuation of the direct vascular response to hypercapnic challenge, further supporting the presence of cerebrovascular dysfunction in this model. Our finding that AD mice had decreased CO_2_ reactivity compared to controls aligns with similar studies in both humans^6,48-50^ and rodents^45,51,52^ which observed a decline in CO_2_ reactivity. Hypercapnia-induced vasodilation in the arteries acts directly on potassium channel activation on vSMCs^53^ or indirectly via vasodilators signalled by endothelial cells, such as nitric oxide.^54^ Indeed, Aβ pathology has been shown to impair potassium channel-related vasodilation in vSMCs^55^ and endothelial function^56^ in AD mice. These impairments may contribute diminished vascular response observed during neurovascular coupling. Additionally, Aβ deposition has been linked to cell-specific dysfunctions within the coordinated neurovascular response, impacting neurons, astrocytes and pericytes.^8^ Collectively, these findings emphasize the importance of early vascular interventions targeted to the individual based on age, sex and underlying pathology.

We observed higher CO_2_ reactivity in control females compared to males across age, but this effect was diminished in AD mice. These finding align with previous studies in humans^28-30^ and rodents^57,58^ which report higher vascular reactivity in healthy females. In humans, CO_2_ reactivity decreases after menopause for females^17-20^ and a recent mouse study reported worse cerebrovascular function after chemically induced menopause.^59^ These observed sex-specific differences are thought to be linked to the steroid hormone expression, as both sexes express oestrogen and androgen receptors on vSMCs and endothelial cells throughout their lifetime.^22^ The robust binding of oestrogen to the vascular endothelium promotes vasodilation through increased production of vasodilators such as nitric oxide,^23,26^ prostacyclins,^24^ hydrogen sulphate signaling^60^ and prostaglandin I2.^22,61^ Additionally, one study reported female mice exhibit greater endothelial-dependent vasodilation and cerebrovascular aromatase expression compared to males, with aromatase inhibition eliminating these sex differences.^58^ Despite the observed sex differences in control mice, AD mice of both sexes showed similarly reduced CO_2_ reactivity by OIS BOLD. Several studies report that oestrogen treatment offers protective effects under normal physiological conditions but may exacerbate certain vasoinflammatory disease states related to ageing and AD.^62-64^ A potential molecular pathway that oestrogen may exacerbate Aβ-induced cerebrovascular damage is through the increased production of nitric oxide in endothelial cells, leading nitrotyrozination of enzymes critical for metabolism and cytoskeletal integrity.^65^ Moreover, sex differences in neuroinflammation observed in ageing and AD result from both independent and interactive effects of sex steroid hormones and sex chromosomes, potentially contributing to the vascular dysfunction seen in this study.^21^ Taken together, our findings suggest that increased Aβ pathology impacts oestrogen’s influence on cerebrovascular function in the AD females. These findings further highlight the potential limitations of oestrogen therapy in older and/or less healthy females.

Our observation that, during hypercapnia, small arterial vessels consistently show greater dilation than large arteries align with previous studies.^32,33^ Interestingly, we observed that AD males exhibited reduced dilation in large arterial vessels, while AD females showed greater impairment in small arteries. These findings appear to align with human studies, where we and others have shown that older females, particularly post-menopausal, compared to older males have increased severity of cerebral small vessel disease burden, as seen through white matter hyperintensities and cortical microbleeds.^13-16^ In contrast, older males may have a higher burden of pathologies associated with the large arteries, such as lacunar infarcts.^15,66,67^ A recent study of APP/PS1 mice found that induced multifocal microinfarcts accelerated AD pathology more potently in males than females, highlighting the partial sex-specificity of vascular dysfunction and pathology in AD at different points in the vasculature.^68^ At the cellular level, sex differences in cerebrovascular structure, inflammation and repair may contribute to these findings. Both androgens and oestrogen promote several processes related to angiogenesis and cerebrovascular repair of the small arterial vessels, partly by increasing vascular endothelial growth factor production.^22,69^ In large arteries, oestrogens appear to protect against arterial stiffening whereas androgens may have deleterious effects.^70^ Additionally, the neuroinflammatory response to ischaemia or vascular insult is partially sex-specific.^71,72^ For example, oestrogen has been attributed to play a cytoprotective role in ischaemic stroke,^73,74^ and clinical evidence demonstrates a higher stroke incidence and severity in females after menopause.^75^ Combined, our findings on sex-specific cerebrovascular dysfunction related to Aβ deposition have important clinical implications for understanding the sex differences in the cerebral small vessel disease biomarkers observed in AD.

A strength of our is study was the ability to measure *in vivo* Aβ tissue plaque and CAA deposition concomitantly with optical imaging assessments of neurovascular and vascular function. We observed that CAA was more strongly associated with diminished CO_2_ reactivity compared to tissue plaque deposition. Research on how AD pathology differentially interacts with the inherent sex differences in vascular function has been limited. Notably, a recent study reported more severe CAA and subsequent microhaemorrhages in female APP/PS1 mice compared to males.^68^ In the context of recent AD clinical trials, CAA has been implicated as a potential driver of the high rates of amyloid-related imaging abnormalities due to Aβ immunotherapy,^76^ and drug efficacy of Aβ immunotherapies may be lower in females compared to males.^77^ Our findings highlight a critical need for future research to characterize the sex-specific effects of CAA and tissue plaque deposition on cerebrovascular pathology and dysfunction, with important implications for the development of personalized diagnostics and therapeutics.

A surprising finding was that neuronal activation in the somatosensory cortex following whisker stimulation trended to increase with age exclusively in AD male mice. AD rodent studies commonly report neuronal hyperactivity in both the hippocampus and cortex, observed at rest^78,79^ and during sensory-evoked stimulation.^80^ Similarly, human functional MRI studies have shown that both the hippocampus and cortex are hyperactive and hyperconnected in early stages when high Aβ deposition is present,^81^ followed by a transition to hypoactivity and hypoconnectivity in later stages of the disease.^82^ However, this transition to hypoactivity is not consistently observed in AD mouse models, where most studies fail to report a shift from neuronal hyper-to hypoexcitability.^83^ Consistent with this, we observed AD male mice exhibited increasing neuronal hyperactivity in the somatosensory cortex with age, with no shift towards hypoexcitation. The majority of AD mouse studies have either used only male mice or included too few females, which may explain the lack of reports on male-specific neuronal hyperexcitability. Providing further evidence for sex-specific, Aβ-driven neuronal effects, we previously demonstrated in humans that sex differentially alters hippocampal connectivity in the presence of increased Aβ burden, with males increasing hippocampal-cortical connectivity as Aβ deposits, while females exhibit no significant change.^84,85^ Together, our findings of a trend that AD male mice, but not females, showed increased neuronal hyperactivity with age underscore the importance of considering sex differences in future AD mouse research.

Several important limitations of this study should be considered. First, we did not measure circulating sex hormone levels or markers of menopause in the mice. However, it is well-documented that mice begin to experience irregular oestrous cycles around 9–12 months of age,^86^ which coincides with the onset of small arterial dysfunction observed in female AD mice. Future research is warranted to directly examine the role of sex steroid hormones in neurovascular and vascular function across ageing. Second, widefield optical imaging measures relative changes from baseline for OIS BOLD, CBV, and GCaMP signals. Variability in baseline physiological measures (e.g. cerebral blood flow) across genotypes and age groups could influence these relative responses, potentially introducing bias. Third, we did not perform longitudinal feature-based tracking of CAA and Aβ tissue plaque volumes. Instead, we averaged measures within each imaging session, which may reduce sensitivity in detecting subtle pathological progression. Although we controlled for sex in all linear regression models, moderate sex differences in plaque burden and CAA vessel coverage were observed and may still influence our findings. Although no sex differences were observed in the diminished OIS BOLD:GCaMP ratio with age among AD mice, the neuronal hyperexcitability identified in AD males may partially contribute to these results. Furthermore, we did not measure features of the time-series besides peak amplitude. Future studies are warranted to examine how the observed neuronal hyperexcitability and diminished NVC response might influence the shape and timing of neurovascular response curves. Finally, the hypercapnia protocol may have introduced behavioural confound. CO_2_ is a known trigger of anxiety in mice,^87^ and previous studies have documented challenges in training awake mice to remain motionless during hypercapnic exposure.^9,88^ Although we excluded imaging time points with excessive motion, the stress-induced state of the mice may have introduced variability or bias into our cerebrovascular measurements.

In summary, our near-lifespan longitudinal study uncovered significant age and sex-specific differences in vascular dysfunction in an APP/PS1 Ad mouse model. AD mice exhibited diminished vascular responses with age, both during neurovascular coupling and hypercapnic conditions. Additionally, while control females maintained higher CO_2_ reactivity compared to males, this advantage was lost in AD female mice. When probing single-vessel dilatory dysfunction, AD females exhibited decreases in small arterial dilation, whereas AD males experienced a diminished dilatory response in larger arterial vessels. Our findings identify sex-specific patholophysiologies of vascular dysfunction in the presence of Aβ pathology, with important implications for developing biomarkers and therapies targeting Aβ-related dysfunction in AD. These findings further underscore the necessity of incorporating sex differences in preclinical AD studies.

## Supplementary Material

fcaf472_Supplementary_Data

## Data Availability

The datasets generated during and/or analysed in the current study are available from the corresponding author upon reasonable request. The code used for data processing and generation of Figs 2–4 is available at: https://github.com/TILabPitt/BrainCommFigures_2025.

## References

[1] Jack CR Jr, Knopman DS, Jagust WJ, et al Tracking pathophysiological processes in Alzheimer’s disease: An updated hypothetical model of dynamic biomarkers. Lancet Neurol. 2013;12(2):207–216. 23332364 10.1016/S1474-4422(12)70291-0 PMC3622225

[2] Iturria-Medina Y, Sotero RC, Toussaint PJ, Mateos-Perez JM, Evans AC, The Alzheimer’s Disease Neuroimaging Initiative. Early role of vascular dysregulation on late-onset Alzheimer’s disease based on multifactorial data-driven analysis. Nat Commun. 2016;7:11934. 27327500 10.1038/ncomms11934 PMC4919512

[3] Sweeney MD, Montagne A, Sagare AP, et al Vascular dysfunction—The disregarded partner of Alzheimer's disease. Alzheimer’s Dement. 2019;15(1):158–167. 30642436 10.1016/j.jalz.2018.07.222 PMC6338083

[4] Greenberg SM, Bacskai BJ, Hernandez-Guillamon M, Pruzin J, Sperling R, van Veluw SJ. Cerebral amyloid angiopathy and Alzheimer disease — One peptide, two pathways. Nat Rev Neurol. 2020;16(1):30–42. 31827267 10.1038/s41582-019-0281-2 PMC7268202

[5] Suri S, Mackay CE, Kelly ME, et al Reduced cerebrovascular reactivity in young adults carrying the *APOE* ε4 allele. Alzheimer’s Dement. 2015;11(6):648–57.e1. 25160043 10.1016/j.jalz.2014.05.1755

[6] Aslanyan V, Mack WJ, Ortega NE, et al Cerebrovascular reactivity in Alzheimer’s disease signature regions is associated with mild cognitive impairment in adults with hypertension. Alzheimer’s Dement. 2024;20(3):1784–1796. 38108158 10.1002/alz.13572 PMC10984494

[7] Alsop DC, Detre JA, Grossman M. Assessment of cerebral blood flow in Alzheimer's disease by spin-labeled magnetic resonance imaging. Ann Neurol. 2000;47(1):93–100. 10632106

[8] Schaeffer S, Iadecola C. Revisiting the neurovascular unit. Nat Neurosci. 2021;24(9):1198–1209. 34354283 10.1038/s41593-021-00904-7 PMC9462551

[9] Tournissac M, Chaigneau E, Pfister S, et al Neurovascular coupling and CO_2_ interrogate distinct vascular regulations. Nat Commun. 2024;15(1):7635. 39223128 10.1038/s41467-024-49698-9 PMC11369082

[10] 2024 Alzheimer’s disease facts and figures. Alzheimer’s Dement. 2024;20(5):3708–3821. 38689398 10.1002/alz.13809 PMC11095490

[11] Nebel RA, Aggarwal NT, Barnes LL, et al Understanding the impact of sex and gender in Alzheimer’s disease: A call to action. Alzheimer’s Dement. 2018;14(9):1171–1183. 29907423 10.1016/j.jalz.2018.04.008 PMC6400070

[12] Ferretti MT, Iulita MF, Cavedo E, et al Sex differences in Alzheimer disease — The gateway to precision medicine. Nat Rev Neurol. 2018;14(8):457–469. 29985474 10.1038/s41582-018-0032-9

[13] Lohner V, Pehlivan G, Sanroma G, et al Relation between sex, menopause, and white matter hyperintensities: The rhineland study. Neurology. 2022;99(9):e935–e943. 35768207 10.1212/WNL.0000000000200782 PMC9502737

[14] Jiménez-Sánchez L, Hamilton OKL, Clancy U, et al Sex differences in cerebral small vessel disease: A systematic review and meta-analysis. Front Neurol. 2021;12:756887. 34777227 10.3389/fneur.2021.756887 PMC8581736

[15] Vinke EJ, de Groot M, Venkatraghavan V, et al Trajectories of imaging markers in brain aging: The rotterdam study. Neurobiol Aging. 2018;71:32–40. 30077040 10.1016/j.neurobiolaging.2018.07.001

[16] Schweitzer N, Son SJ, Thurston RC, et al Sex-specific risk factors and clinical dementia outcomes for white matter hyperintensities in a large south Korean cohort. Alzheimers Res Ther. 2024;16(1):243. 39482724 10.1186/s13195-024-01598-2 PMC11529246

[17] Moir ME, Corkery AT, Senese KA, et al Age at natural menopause impacts cerebrovascular reactivity and brain structure. Am J Physiol Regul Integr Comp Physiol. 2023;324(2):R207–R215. 36622085 10.1152/ajpregu.00228.2022 PMC9886341

[18] Skinner BD, Davies RJ, Weaver SR, Cable NT, Lucas SJE, Lucas RAI. A systematic review and meta-analysis examining whether changing ovarian sex steroid hormone levels influence cerebrovascular function. Front Physiol. 2021;12:687591. 34220552 10.3389/fphys.2021.687591 PMC8248489

[19] Matteis M, Troisi E, Monaldo BC, Caltagirone C, Silvestrini M. Age and sex differences in cerebral hemodynamics: A transcranial Doppler study. Stroke. 1998;29(5):963–967. 9596243 10.1161/01.str.29.5.963

[20] Barnes JN, Harvey RE, Eisenmann NA, et al Cerebrovascular reactivity after cessation of menopausal hormone treatment. Climacteric. 2019;22(2):182–189. 30661405 10.1080/13697137.2018.1538340 PMC6428595

[21] Lopez-Lee C, Torres ERS, Carling G, Gan L. Mechanisms of sex differences in Alzheimer’s disease. Neuron. 2024;112(8):1208–1221. 38402606 10.1016/j.neuron.2024.01.024 PMC11076015

[22] Robison LS, Gannon OJ, Salinero AE, Zuloaga KL. Contributions of sex to cerebrovascular function and pathology. Brain Res. 2019;1710:43–60. 30580011 10.1016/j.brainres.2018.12.030

[23] Chen Z, Yuhanna IS, Galcheva-Gargova Z, Karas RH, Mendelsohn ME, Shaul PW. Estrogen receptor α mediates the nongenomic activation of endothelial nitric oxide synthase by estrogen. J Clin Invest. 1999;103(3):401–406. 9927501 10.1172/JCI5347 PMC407904

[24] Egan KM, Lawson JA, Fries S, et al COX-2-derived prostacyclin confers atheroprotection on female mice. Science. 2004;306(5703):1954–1957. 15550624 10.1126/science.1103333

[25] Scotland RS, Madhani M, Chauhan S, et al Investigation of vascular responses in endothelial nitric oxide synthase/cyclooxygenase-1 double-knockout mice. Circulation. 2005;111(6):796–803. 15699263 10.1161/01.CIR.0000155238.70797.4E

[26] Geary GG, Krause DN, Duckles SP. Estrogen reduces mouse cerebral artery tone through endothelial NOS- and cyclooxygenase-dependent mechanisms. Am J Physiol Heart Circ Physiol. 2000;279(2):H511–H519. 10924048 10.1152/ajpheart.2000.279.2.H511

[27] Russell KS, Haynes MP, Sinha D, Clerisme E, Bender JR. Human vascular endothelial cells contain membrane binding sites for estradiol, which mediate rapid intracellular signaling. Proc Natl Acad Sci U S A. 2000;97(11):5930–5935. 10823945 10.1073/pnas.97.11.5930 PMC18536

[28] Kastrup A, Dichgans J, Niemeier M, Schabet M. Changes of cerebrovascular CO_2_ reactivity during normal aging. Stroke. 1998;29(7):1311–1314. 9660378 10.1161/01.str.29.7.1311

[29] Kastrup A, Thomas C, Hartmann C, Schabet M. Sex dependency of cerebrovascular CO_2_ reactivity in normal subjects. Stroke. 1997;28(12):2353–2356. 9412613 10.1161/01.str.28.12.2353

[30] Deegan BM, Sorond FA, Galica A, Lipsitz LA, O'Laighin G, Serrador JM. Elderly women regulate brain blood flow better than men do. Stroke. 2011;42(7):1988–1993. 21566238 10.1161/STROKEAHA.110.605618 PMC7111558

[31] Nicholson CJ, Sweeney M, Robson SC, Taggart MJ. Estrogenic vascular effects are diminished by chronological aging. Sci Rep. 2017;7(1):12153. 28939871 10.1038/s41598-017-12153-5 PMC5610317

[32] Iadecola C, Yang G, Ebner TJ, Chen G. Local and propagated vascular responses evoked by focal synaptic activity in cerebellar Cortex. J Neurophysiol. 1997;78(2):651–659. 9307102 10.1152/jn.1997.78.2.651

[33] Lee S-P, Duong TQ, Yang G, Iadecola C, Kim S-G. Relative changes of cerebral arterial and venous blood volumes during increased cerebral blood flow: Implications for BOLD fMRI. Magn Reson Med. 2001;45(5):791–800. 11323805 10.1002/mrm.1107

[34] Jankowsky JL, Slunt HH, Gonzales V, Jenkins NA, Copeland NG, Borchelt DR. APP processing and amyloid deposition in mice haplo-insufficient for presenilin 1. Neurobiol Aging. 2004;25(7):885–892. 15212842 10.1016/j.neurobiolaging.2003.09.008

[35] Zhong MZ, Peng T, Duarte ML, Wang M, Cai D. Updates on mouse models of Alzheimer’s disease. Mol Neurodegener. 2024;19(1):23. 38462606 10.1186/s13024-024-00712-0 PMC10926682

[36] Jiao S-S, Bu X-L, Liu Y-H, et al Sex dimorphism profile of Alzheimer’s disease-type pathologies in an APP/PS1 mouse model. Neurotox Res. 2016;29:256–266. 26707129 10.1007/s12640-015-9589-x

[37] Krawchuk MB, Ruff CF, Yang X, Ross SE, Vazquez AL. Optogenetic assessment of VIP, PV, SOM and NOS inhibitory neuron activity and cerebral blood flow regulation in mouse somato-sensory cortex. J Cereb Blood Flow Metab. 2020;40(7):1427–1440. 31418628 10.1177/0271678X19870105 PMC7307010

[38] Paxinos G, Franklin KB. Paxinos and franklin's the mouse brain in stereotaxic coordinates. Academic press; 2019.

[39] D’Alonzo KT . The johnson-neyman procedure as an alternative to ANCOVA. West J Nurs Res. 2004;26(7):804–812. 15466616 10.1177/0193945904266733 PMC3203541

[40] Hoiland RL, Fisher JA, Ainslie PN. Regulation of the cerebral circulation by arterial carbon dioxide. Compr Physiol. 2011;9(3):1101–1154. 10.1002/cphy.c180021 31187899

[41] Morikawa T, Kajimura M, Nakamura T, et al Hypoxic regulation of the cerebral microcirculation is mediated by a carbon monoxide-sensitive hydrogen sulfide pathway. Proc Natl Acad Sci U S A. 2012;109(4):1293–1298. 22232681 10.1073/pnas.1119658109 PMC3268316

[42] Park L, Koizumi K, El Jamal S, et al Age-dependent neurovascular dysfunction and damage in a mouse model of cerebral amyloid angiopathy. Stroke. 2014;45(6):1815–1821. 24781082 10.1161/STROKEAHA.114.005179 PMC4284427

[43] Van Skike CE, Hussong SA, Hernandez SF, Banh AQ, DeRosa N, Galvan V. mTOR attenuation with rapamycin reverses neurovascular uncoupling and memory deficits in mice modeling Alzheimer’s disease. J Neurosci. 2021;41(19):4305–4320. 33888602 10.1523/JNEUROSCI.2144-20.2021 PMC8143195

[44] Lourenço CF, Ledo A, Barbosa RM, Laranjinha J. Neurovascular uncoupling in the triple transgenic model of Alzheimer’s disease: Impaired cerebral blood flow response to neuronal-derived nitric oxide signaling. Exp Neurol. 2017;291:36–43. 28161255 10.1016/j.expneurol.2017.01.013

[45] Shin HK, Jones PB, Garcia-Alloza M, et al Age-dependent cerebrovascular dysfunction in a transgenic mouse model of cerebral amyloid angiopathy. Brain. 2007;130(9):2310–2319. 17638859 10.1093/brain/awm156

[46] Vestergaard MB, Bakhtiari A, Osler M, et al The cerebral blood flow response to neuroactivation is reduced in cognitively normal men with β-amyloid accumulation. Alzheimers Res Ther. 2025;17(1):4. 39754275 10.1186/s13195-024-01652-z PMC11699738

[47] van Dijk SE, Drenth N, Hafkemeijer A, et al Neurovascular coupling in early stage dementia—A case-control study. J Cereb Blood Flow Metab. 2024;44(6):1013–1023. 37994030 10.1177/0271678X231214102 PMC11318393

[48] Alwatban M, Murman DL, Bashford G. Cerebrovascular reactivity impairment in preclinical Alzheimer’s disease. J Neuroimaging. 2019;29(4):493–498. 30748053 10.1111/jon.12606

[49] Richiardi J, Monsch AU, Haas T, et al Altered cerebrovascular reactivity velocity in mild cognitive impairment and Alzheimer’s disease. Neurobiol Aging. 2015;36(1):33–41. 25146454 10.1016/j.neurobiolaging.2014.07.020

[50] Sur S, Lin Z, Li Y, et al Association of cerebrovascular reactivity and Alzheimer pathologic markers with cognitive performance. Neurology. 2020;95(8):e962–e972. 32661101 10.1212/WNL.0000000000010133 PMC7668551

[51] Ongali B, Nicolakakis N, Lecrux C, et al Transgenic mice overexpressing APP and transforming growth factor-β1 feature cognitive and vascular hallmarks of Alzheimer’s disease. Am J Pathol. 2010;177(6):3071–3080. 21088218 10.2353/ajpath.2010.100339 PMC2993264

[52] Dorr A, Sahota B, Chinta LV, et al Amyloid-β-dependent compromise of microvascular structure and function in a model of Alzheimer’s disease. Brain. 2012;135(10):3039–3050. 23065792 10.1093/brain/aws243

[53] Peng H-L, Jensen PE, Nilsson H, Aalkjær C. Effect of acidosis on tension and [ca^2+^]_i_ in rat cerebral arteries: Is there a role for membrane potential? Am J Physiol Heart Circ Physiol. 1998;274(2):H655–H662. 10.1152/ajpheart.1998.274.2.H655 9486271

[54] Fathi AR, Yang C, Bakhtian KD, Qi M, Lonser RR, Pluta RM. Carbon dioxide influence on nitric oxide production in endothelial cells and astrocytes: Cellular mechanisms. Brain Res. 2011;1386:50–57. 21362408 10.1016/j.brainres.2011.02.066 PMC3073030

[55] Taylor JL, Pritchard HA, Walsh KR, et al Functionally linked potassium channel activity in cerebral endothelial and smooth muscle cells is compromised in Alzheimer’s disease. Proc Natl Acad Sci U S A. 2022;119(26):e2204581119. 35727988 10.1073/pnas.2204581119 PMC9245656

[56] Taylor Jade L, Martin-Aragon Baudel M, Nieves-Cintron M, Navedo Manuel F. Vascular function and Ion channels in Alzheimer’s disease. Microcirculation. 2024;31:e12881. 39190776 10.1111/micc.12881 PMC11498901

[57] Geary GG, Krause DN, Duckles SP. Estrogen reduces myogenic tone through a nitric oxide-dependent mechanism in rat cerebral arteries. Am J Physiol Heart Circ Physiol. 1998;275(1):H292–H300. 10.1152/ajpheart.1998.275.1.H292 9688926

[58] Zuloaga KL, Davis CM, Zhang W, Alkayed NJ. Role of aromatase in sex-specific cerebrovascular endothelial function in mice. Am J Physiol Heart Circ Physiol. 2014;306(7):H929–H937. 24508640 10.1152/ajpheart.00698.2013 PMC3962636

[59] Blackwell JA, Silva JF, Louis EM, et al Cerebral arteriolar and neurovascular dysfunction after chemically induced menopause in mice. Am J Physiol Heart Circ Physiol. 2022;323(5):H845–H860. 36149767 10.1152/ajpheart.00276.2022 PMC9602916

[60] Eberhardt M, Dux M, Namer B, et al H_2_s and NO cooperatively regulate vascular tone by activating a neuroendocrine HNO–TRPA1–CGRP signalling pathway. Nat Commun. 2014;5(1):4381. 25023795 10.1038/ncomms5381 PMC4104458

[61] Ospina JA, Krause DN, Duckles SP. 17β-Estradiol increases rat cerebrovascular prostacyclin synthesis by elevating cyclooxygenase-1 and prostacyclin synthase. Stroke. 2002;33(2):600–605. 11823676 10.1161/hs0202.102732

[62] Marriott L, Hauss-Wegrzyniak B, Benton R, Vraniak P, Wenk G. Long-term estrogen therapy worsens the behavioral and neuropathological consequences of chronic brain inflammation. Behav Neurosci. 2002;116(5):902. 12369809 10.1037//0735-7044.116.5.902

[63] Mukherjee TK, Reynolds PR, Hoidal JR. Differential effect of estrogen receptor alpha and beta agonists on the receptor for advanced glycation end product expression in human microvascular endothelial cells. Biochim Biophys Acta. 2005;1745(3):300–309. 15878629 10.1016/j.bbamcr.2005.03.012

[64] Xu H-L, Baughman VL, Pelligrino DA. Estrogen replacement treatment in diabetic ovariectomized female rats potentiates postischemic leukocyte adhesion in cerebral venules. Stroke. 2004;35(8):1974–1978. 15232125 10.1161/01.STR.0000135016.24349.9F

[65] Coma M, Guix FX, Uribesalgo I, et al Lack of oestrogen protection in amyloid-mediated endothelial damage due to protein nitrotyrosination. Brain. 2005;128(7):1613–1621. 15817516 10.1093/brain/awh492

[66] Exalto LG, Boomsma JMF, Babapour Mofrad R, et al Sex differences in memory clinic patients with possible vascular cognitive impairment. Alzheimers Dement (Amst). 2020;12(1):e12090. 32875057 10.1002/dad2.12090 PMC7447910

[67] Oveisgharan S, Arvanitakis Z, Yu L, Farfel J, Schneider JA, Bennett DA. Sex differences in Alzheimer’s disease and common neuropathologies of aging. Acta Neuropathol. 2018;136(6):887–900. 30334074 10.1007/s00401-018-1920-1 PMC6279593

[68] Lecordier S, Pons V, Rivest S, ElAli A. Multifocal cerebral microinfarcts modulate early Alzheimer’s disease pathology in a sex-dependent manner. Front Immunol. 2022;12:813536. 35173711 10.3389/fimmu.2021.813536 PMC8841345

[69] McCullough LD, Hurn PD. Estrogen and ischemic neuroprotection: An integrated view. Trends Endocrinol Metab. 2003;14(5):228–235. 12826329 10.1016/s1043-2760(03)00076-6

[70] Haast RA, Gustafson DR, Kiliaan AJ. Sex differences in stroke. J Cereb Blood Flow Metab. 2012;32(12):2100–2107. 23032484 10.1038/jcbfm.2012.141 PMC3519418

[71] Villa A, Gelosa P, Castiglioni L, et al Sex-specific features of microglia from adult mice. Cell Rep. 2018;23(12):3501–3511. 29924994 10.1016/j.celrep.2018.05.048 PMC6024879

[72] Ugidos IF, Pistono C, Korhonen P, et al Sex differences in poststroke inflammation: A focus on microglia across the lifespan. Stroke. 2022;53(5):1500–1509. 35468000 10.1161/STROKEAHA.122.039138

[73] Rahimian R, Cordeau P Jr, Kriz J. Brain response to injuries: When microglia go sexist. Neuroscience. 2019;405:14–23. 29526689 10.1016/j.neuroscience.2018.02.048

[74] Guo H, Yang J, Liu M, et al Selective activation of estrogen receptor β alleviates cerebral ischemia neuroinflammatory injury. Brain Res. 2020;1726:146536. 31676226 10.1016/j.brainres.2019.146536

[75] Lisabeth L, Bushnell C. Stroke risk in women: The role of menopause and hormone therapy. Lancet Neurol. 2012;11(1):82–91. 22172623 10.1016/S1474-4422(11)70269-1 PMC3615462

[76] Sveikata L, Charidimou A, Viswanathan A. Vessels Sing their ARIAs: The role of vascular amyloid in the age of aducanumab. Stroke. 2022;53(1):298–302. 34905943 10.1161/STROKEAHA.121.036873

[77] Kurkinen M . Lecanemab (leqembi) is not the right drug for patients with Alzheimer’s disease. Adv Clin Exp Med. 2023;32(9):943–947. 37676096 10.17219/acem/171379

[78] Busche MA, Chen X, Henning HA, et al Critical role of soluble amyloid-β for early hippocampal hyperactivity in a mouse model of Alzheimer’s disease. Proc Natl Acad Sci U S A. 2012;109(22):8740–8745. 22592800 10.1073/pnas.1206171109 PMC3365221

[79] Busche MA, Eichhoff G, Adelsberger H, et al Clusters of hyperactive neurons near amyloid plaques in a mouse model of Alzheimer’s disease. Science. 2008;321(5896):1686–1689. 18802001 10.1126/science.1162844

[80] Hefendehl JK, LeDue J, Ko RWY, Mahler J, Murphy TH, MacVicar BA. Mapping synaptic glutamate transporter dysfunction in vivo to regions surrounding aβ plaques by iGluSnFR two-photon imaging. Nat Commun. 2016;7(1):13441. 27834383 10.1038/ncomms13441 PMC5114608

[81] Huijbers W, Mormino EC, Wigman SE, et al Amyloid deposition is linked to aberrant entorhinal activity among cognitively normal older adults. J Neurosci. 2014;34(15):5200–5210. 24719099 10.1523/JNEUROSCI.3579-13.2014 PMC3983800

[82] Machulda MM, Ward HA, Borowski B, et al Comparison of memory fMRI response among normal, MCI, and Alzheimer’s patients. Neurology. 2003;61(4):500–506. 12939424 10.1212/01.wnl.0000079052.01016.78 PMC2744465

[83] Targa Dias Anastacio H, Matosin N, Ooi L. Neuronal hyperexcitability in Alzheimer’s disease: What are the drivers behind this aberrant phenotype? Transl Psychiatry. 2022;12(1):257. 35732622 10.1038/s41398-022-02024-7 PMC9217953

[84] Schweitzer N, Li J, Thurston RC, et al Sex-dependent alterations in hippocampal connectivity are linked to cerebrovascular and amyloid pathologies in normal aging. Alzheimer’s Dement. 2024;20:914–9924. 37817668 10.1002/alz.13503 PMC10916980

[85] Wu M, Thurston RC, Tudorascu DL, et al Amyloid deposition is associated with different patterns of hippocampal connectivity in men versus women. Neurobiol Aging. 2019;76:141–150. 30711677 10.1016/j.neurobiolaging.2018.11.020 PMC6584958

[86] Koebele SV, Bimonte-Nelson HA. Modeling menopause: The utility of rodents in translational behavioral endocrinology research. Maturitas. 2016;87:5–17. 27013283 10.1016/j.maturitas.2016.01.015 PMC4829404

[87] Leibold NK, van den Hove DLA, Viechtbauer W, et al CO_2_ exposure as translational cross-species experimental model for panic. Transl Psychiatry. 2016;6(9):e885–e885. 27598969 10.1038/tp.2016.162 PMC5048202

[88] Hosford PS, Wells JA, Nizari S, et al CO_2_ signaling mediates neurovascular coupling in the cerebral cortex. Nat Commun. 2022;13(1):2125. 35440557 10.1038/s41467-022-29622-9 PMC9019094

