# Supplementary material for: Near-lifespan mesoscopic optical imaging of cerebrovascular function reveals age and sex differences in preclinical Alzheimer’s disease model
Source: Brain Commun. 2025 Dec 3;7(6):fcaf472. doi: 10.1093/braincomms/fcaf472 (PMC12699225; doi:10.1093/braincomms/fcaf472)
Supplement: fcaf472_Supplementary_Data [file fcaf472_supplementary_data.docx]

**Supplementary Material**

**Supplementary Tables**

**Supplementary Table 1. Linear mixed effect regression analyses of peak GCaMP response against age for the whisker puffing stimulus. *Related to Main Figure 3.***

| **Peak Neuronal Response vs. Age Response Linear Mixed Effect Regression Results**  **Model 1: GCaMP ~ Age + Sex + (1\|Subject)**  **Model 2: GCaMP ~ Age + Sex + Age×Sex + (1\|Subject)** | | | |
| --- | --- | --- | --- |
| **Cohort** | **Model 1: Age effect**  **B (SE),**  **p-values** | **Model 1: Sex effect**  **B (SE),**  **p-values** | **Model 2: Age*Sex,**  **B (SE),**  **p-values** |
| AD Mice | 0.047 (0.041),  *p* = 0.26 | 0.024 (0.55),  *p* = 0.97 | 0.14 (0.082),  *p* = 0.092 |
| WT Mice | -0.040 (0.049),  *p* = 0.42 | 1.16 (0.68),  *p* = 0.14 | -0.083 (0.098),  *p* = 0.40 |

**p*< 0.05; ***p*< 0.01; ****p*< 0.001

N = 77, 68 for AD and WT mice, respectively.

GCaMP = GCaMP6f fluorescent signal.

**Supplementary Table 2. Linear mixed effect regression analyses of peak GCaMP response against Aβ pathology for the whisker puffing stimulus.**

| **Peak Neuronal Response vs. Age Response Linear Mixed Effect Regression Results**  **Model 1: GCaMP ~ CAA vessel coverage + (1\|Subject)**  **Model 2: GCaMP ~ log Aβ tissue plaque volume + (1\|Subject)**  **Model 3: GCaMP ~ Sex + log Aβ plaque + Sex×log Aβ plaque + (1\|Subject)**  **Model 4: GCaMP ~ Sex + CAA + Sex×CAA + (1\|Subject)** | | | | |
| --- | --- | --- | --- | --- |
| **Cohort** | **Model 1: Aβ tissue plaque effect**  **B (SE),**  **p-values** | **Model 2: CAA effect**  **B (SE),**  **p-values** | **Model 3: Sex*Aβ plaque effect**  **B (SE),**  **p-values** | **Model 4: Sex*CAA effect**  **B (SE),**  **p-values** |
| AD Males | 0.31 (0.30),  *p* = 0.34 | 0.053 (0.04),  *p* = 0.22 | 0.44 (0.34),  *p* = 0.20 | 0.098 (0.053),  *p* = 0.076 |
| AD Females | -0.12 (0.18),  *p* = 0.49 | -0.043 (0.030),  *p* =0.18 | 0.44 (0.34),  *p* = 0.20 | 0.098 (0.053),  *p* = 0.076 |

**p*< 0.05; ***p*< 0.01; ****p*< 0.001

N = 58, 59 for CAA and Aβ tissue plaque analyses, respectively.

CAA = Cerebral Amyloid Angiopathy; GCaMP = GCaMP6f fluorescent signal.

**Supplementary Table 3. Linear mixed effect regression analyses of OIS BOLD vs GCaMP response for the whisker puffing stimulus. *Related to Main Figure 3.***

| **OIS BOLD vs GCaMP Response Linear Mixed Effect Regression Results**  **Model 1: OIS BOLD ~ GCaMP + Sex + (1\|Subject)**  **Model 2: OIS BOLD ~ GCaMP + Sex + Genotype + (1\|Subject)** | | | |
| --- | --- | --- | --- |
| **Cohort** | **Model 1: GCaMP effect**  **B (SE),**  **p-values** | **Model 1: Sex effect**  **B (SE),**  **p-values** | **Model 2: Genotype,**  **B (SE),**  **p-values** |
| AD Mice | 0.050 (0.01),  *p* = 3.8E-4*** | 0.0086 (0.04),  *p* = 0.84 | 0.077 (0.036),  *p* = 0.050* |
| WT Mice | 0.037 (0.15),  *p* = 0.018** | 0.093 (0.06),  *p* = 0.20 | 0.077 (0.036),  *p* = 0.050* |

**p*< 0.05; ***p*< 0.01; ****p*< 0.001

N = 77, 68 for AD and WT mice, respectively.

OIS-BOLD = deoxy-hemoglobin-weighted (blood oxygen level dependent) optical intrinsic signal; GCaMP = GCaMP6f fluorescent signal.

**Supplementary Table 4. Linear mixed effect regression analyses testing the association of AD pathology and age on BOLD:GCaMP ratio for the whisker puffing stimulus. *Related to Main Figure 3.***

| **OIS BOLD:GCaMP Response Linear Mixed Effect Regression Results**  **Model 1: OIS BOLD:GCaMP ~ Age + Sex + (1\|Subject)**  **Model 2: OIS BOLD:GCaMP ~ Sex + CAA vessel coverage + (1\|Subject)**  **Model 3: OIS BOLD:GCaMP ~ Sex + log Aβ tissue plaque volume + (1\|Subject)** | | | | |
| --- | --- | --- | --- | --- |
| **Cohort** | **Model 1: Age effect**  **B (SE),**  **p-values** | **Model 1: Sex effect**  **B (SE),**  **p-values** | **Model 2: CAA effect**  **B (SE),**  **p-values** | **Model 3: Plaque effect**  **B (SE),**  **p-values** |
| AD Mice | -0.0047 (0.0016),  *p* = 0.0051* | 0.0054 (0.014),  *p* = 0.71 | -0.0012 (0.0010),  *p* = 0.23 | -0.0013 (0.006),  *p* = 0.84 |
| WT Mice | -0.00018 (0.0025),  *p* = 0.95 | 0.0053 (0.028),  *p* = 0.85 | N/A | N/A |

**p*< 0.05; ***p*< 0.01; ****p*< 0.001

N = 58, 59 for analyses on tissue plaque volume and CAA, respectively.

OIS-BOLD = deoxy-hemoglobin-weighted (blood oxygen level dependent) optical intrinsic signal; GCaMP = GCaMP6f fluorescent signal; CAA = Cerebral Amyloid Angiopathy

**Supplementary Table 5: Linear mixed effect regression analyses to test the association of age and sex on OIS BOLD and CBV response for the hypercapnia challenge. *Related to Main Figure 5.***

| **OIS BOLD Response Linear Mixed Effect Regression Results**  **Model 1: OIS BOLD ~ Age + Sex + (1\|Subject)**  **Model 2: OIS BOLD ~ Age + Genotype + Age×Genotype + (1\|Subject)** | | | |
| --- | --- | --- | --- |
| **Cohort** | **Model 1: Age effect**  **B (SE),**  **p-values** | **Model 1: Sex effect**  **B (SE),**  **p-values** | **Model 2: Age*Genotype,**  **B (SE),**  **p-values** |
| AD Mice | -0.067 (0.020),  *p* = 0.0011** | -0.35 (0.21),  *p* = 0.11 | 0.064 (0.030),  *p* = 0.036* |
| WT Mice | -0.024 (0.02),  *p* = 0.25 | -0.65 (0.30),  *p* = 0.043* | 0.064 (0.030),  *p* = 0.036* |
| **OIS CBV Response Linear Mixed Effect Regression Results**  **Model 1: OIS CBV ~ Age + Sex + (1\|Subject)**  **Model 2: OIS CBV ~ Age + Genotype + Age×Genotype + (1\|Subject)** | | | |
| **Cohort** | **Model 1: Age effect**  **B (SE),**  **p-values** | **Model 1: Sex effect**  **B (SE),**  **p-values** | **Model 2: Age*Genotype,**  **B (SE),**  **p-values** |
| AD Mice | -0.25 (0.099),  *p* = 0.015* | -0.98 (1.1),  *p* = 0.39 | 0.42 (0.14),  *p* = 0.0029* |
| WT Mice | 0.19 (0.11),  *p* = 0.078 | -0.59 (0.81),  *p* = 0.49 | 0.41 (0.13),  *p* = 0. 0029* |

**p*< 0.05; ***p*< 0.01; ****p*< 0.001

N = 108, 97 for AD and WT mice, respectively.

OIS-BOLD = deoxy-hemoglobin-weighted (blood oxygen level dependent) optical intrinsic signal; CBV = total hemoglobin-weighted (cerebral blood volume) optical intrinsic signal.

**Supplementary Table 6: Linear mixed effect regression analyses of OIS BOLD and CBV response versus AD pathology for the hypercapnia challenge. *Related to Main Figure 5.***

| **OIS BOLD Response Linear Mixed Effect Regression Results**  **Model 1: OIS BOLD ~ CAA + Sex + (1\|Subject)**  **Model 2: OIS BOLD ~ log Aβ plaque + Sex + (1\|Subject)** | | |
| --- | --- | --- |
| **Cohort** | **Model 1: CAA effect**  **B (SE),**  **p-values** | **Model 2: log Aβ plaque effect**  **B (SE),**  **p-values** |
| AD Mice | -0.026 (0.012),  *p* = 0.033* | -0.082 (0.06),  *p* = 0.21 |
| **OIS CBV Response Linear Mixed Effect Regression Results**  **Model 1: OIS CBV ~ CAA + Sex + (1\|Subject)**  **Model 2: OIS CBV ~ log Aβ plaque + Sex + (1\|Subject)** | | |
| **Cohort** | **Model 1: CAA effect**  **B (SE),**  **p-values** | **Model 2: log Aβ plaque effect**  **B (SE),**  **p-values** |
| AD Mice | -0.086 (0.041),  *p* = 0.056 | -0.10 (0.26),  *p* = 0.69 |

**p*< 0.05; ***p*< 0.01; ****p*< 0.001

N = 58, 59 for analyses on tissue plaque volume and CAA, respectively.

OIS-BOLD = deoxy-hemoglobin-weighted (blood oxygen level dependent) optical intrinsic signal; CBV = total hemoglobin-weighted (cerebral blood volume) optical intrinsic signal; CAA = Cerebral Amyloid Angiopathy.

**Supplementary Table 7: Linear mixed effect regression analysis on the effect of age and sex on baseline vessel diameter.**

| **Arterial Diameter Response Linear Mixed Effect Regression Results**  **Model 1: Baseline diameter ~ Age + Sex + (1\|Subject)** | | |
| --- | --- | --- |
| **Cohort** | **Model 1: Age effect**  **B (SE),**  **p-values** | **Model 1: Sex effect**  **B (SE),**  **p-values** |
| AD Mice | 0.13, (0.16),  *p* = 0.43 | 1.11 (2.66),  *p* = 0.68 |
| WT Mice | 0.35 (0.19),  *p* = 0.064 | -5.22 (4.25),  *p* = 0.24 |
| **Venous Diameter Response Linear Mixed Effect Regression Results**  **Model 1: Baseline diameter ~ Age + Sex + (1\|Subject)** | | |
| **Cohort** | **Model 1: Age effect**  **B (SE),**  **p-values** | **Model 1: Sex effect**  **B (SE),**  **p-values** |
| AD Mice | 0.47 (0.22),  *p* = 0.033* | 3.85 (3.45),  *p* = 0.28 |
| WT Mice | 0.42 (0.23),  *p* = 0.075 | -8.63 (5.75),  *p* = 0.16 |

**p*< 0.05; ***p*< 0.01; ****p*< 0.001

N = 697, 689 for AD and WT arterial vessels, respectively. N = 1031, 778 for AD and WT venous vessels, respectively.

**Supplementary Table 8: Linear mixed effect regression analysis to test the effect of age and sex on vessel diameter dilation response during hypercapnia challenge while controlling for baseline diameter. *Related to Main Figure 7.***

| **Arterial Diameter Response Linear Mixed Effect Regression Results**  **Model 1: % diameter change ~ Age + Sex + Baseline diameter + (1\|Subject)**  **Model 2: % diameter change ~ Age + Genotype + Sex + Baseline diameter + Age×Genotype + (1\|Subject)**  **Model 3: % diameter change ~ Age + Genotype + Sex + Baseline diameter + Genotype×Sex + (1\|Subject)** | | | |
| --- | --- | --- | --- |
| **Cohort** | **Model 1: Age effect**  **B (SE),**  **p-values** | **Model 1: Sex effect**  **B (SE),**  **p-values** | **Model 1: Baseline diameter effect**  **B (SE),**  **p-values** |
| AD Mice | -0.29 (0.087),  *p* = 0.0012*** | 0.98 (1.35),  *p* = 0.48 | -0.10 (0.021),  *p* = 1.83E-6*** |
| WT Mice | -0.0057 (0.12),  *p* = 0.96 | 0.42 (1.64),  *p* = 0.80 | -0.21 (0.026),  *p* = 6.9E-15*** |
| **Venous Diameter Response Linear Mixed Effect Regression Results**  **Model 1: % diameter change ~ Age + Sex + Baseline diameter + (1\|Subject)**  **Model 2: % diameter change ~ Age + Genotype + Baseline diameter + Age×Genotype + (1\|Subject)** | | | |
| **Cohort** | **Model 1: Age effect**  **B (SE),**  **p-values** | **Model 1: Sex effect**  **B (SE),**  **p-values** | **Model 1: Baseline diameter effect**  **B (SE),**  **p-values** |
| AD Mice | -0.12 (0.054),  *p* = 0.039* | 1.99 (1.1),  *p* = 0.090 | 0.024 (0.0075),  *p* = 0.0012* |
| WT Mice | 0.17 (0.08),  *p* = 0.035* | 2.14 (1.3),  *p* = 0.13 | 0.031 (0.012),  *p* = 0.012* |

**p*< 0.05; ***p*< 0.01; ****p*< 0.001

N = 697, 689 for AD and WT arterial vessels, respectively. N = 1031, 778 for AD and WT venous vessels, respectively.

**Supplementary Table 9: Linear mixed effect regression analysis to test the effect of AD pathology with small and large arterial diameter dilation response during hypercapnia challenge. *Related to Main Figure 9.***

| **Small Arterial Diameter Response Linear Mixed Effect Regression Results**  **Model 1: % diameter change ~ log Aβ plaque + Baseline diameter + (1\|Subject)**  **Model 2: % diameter change ~ CAA vessel coverage + Baseline diameter + (1\|Subject)** | | |
| --- | --- | --- |
| **Cohort** | **Model 1: Aβ plaque effect**  **B (SE),**  **p-values** | **Model 2: CAA effect**  **B (SE),**  **p-values** |
| AD Male | -0.12 (0.74),  *p* = 0.88 | -0.13 (0.12),  *p* = 0.36 |
| AD Female | -1.31 (0.53),  *p* = 0.016** | -0.33 (0.12),  *p* = 0.0062** |
| **Large Arterial Diameter Response Linear Mixed Effect Regression Results**  **Model 1: % diameter change ~ log Aβ plaque + Baseline diameter + (1\|Subject)**  **Model 2: % diameter change ~ CAA vessel coverage + Baseline diameter + (1\|Subject)** | | |
| **Cohort** | **Model 1: Aβ effect**  **B (SE),**  **p-values** | **Model 2: CAA effect**  **B (SE),**  **p-values** |
| AD Male | 2.07 (1.2),  *p* = 0.13 | -0.065 (0.16),  *p* = 0.70 |
| AD Female | -0.20 (0.32),  *p* = 0.54 | -0.0074 (0.08),  *p* = 0.93 |

**p*< 0.05; ***p*< 0.01; ****p*< 0.001

N = 161, N = 158 for CAA and tissue plaque volume analyses respectively.

CAA = Cerebral Amyloid Angiopathy

**Supplementary Figures**


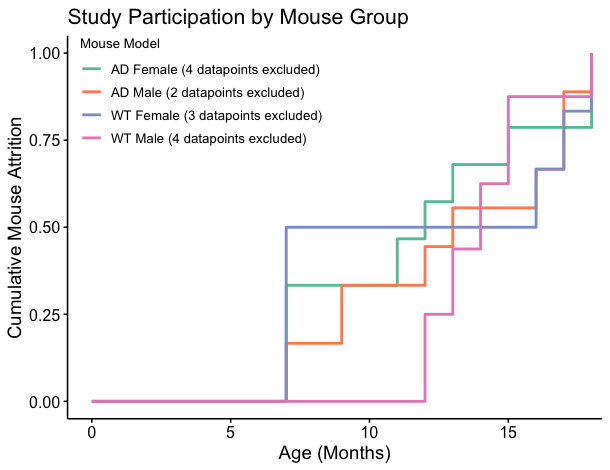


**Supplementary Figure 1. Cumulative attrition of mice by age for each genotype group.** Each group began with N = 8 mice. Notably, all groups retained at least 4 mice until 12 months of age. On average, mice were imaged at 5.4 ± 3.1 time points, with a mean interval 1.52 ± 0.5 months between imaging sessions. Datapoints excluded were due to excessive motion.


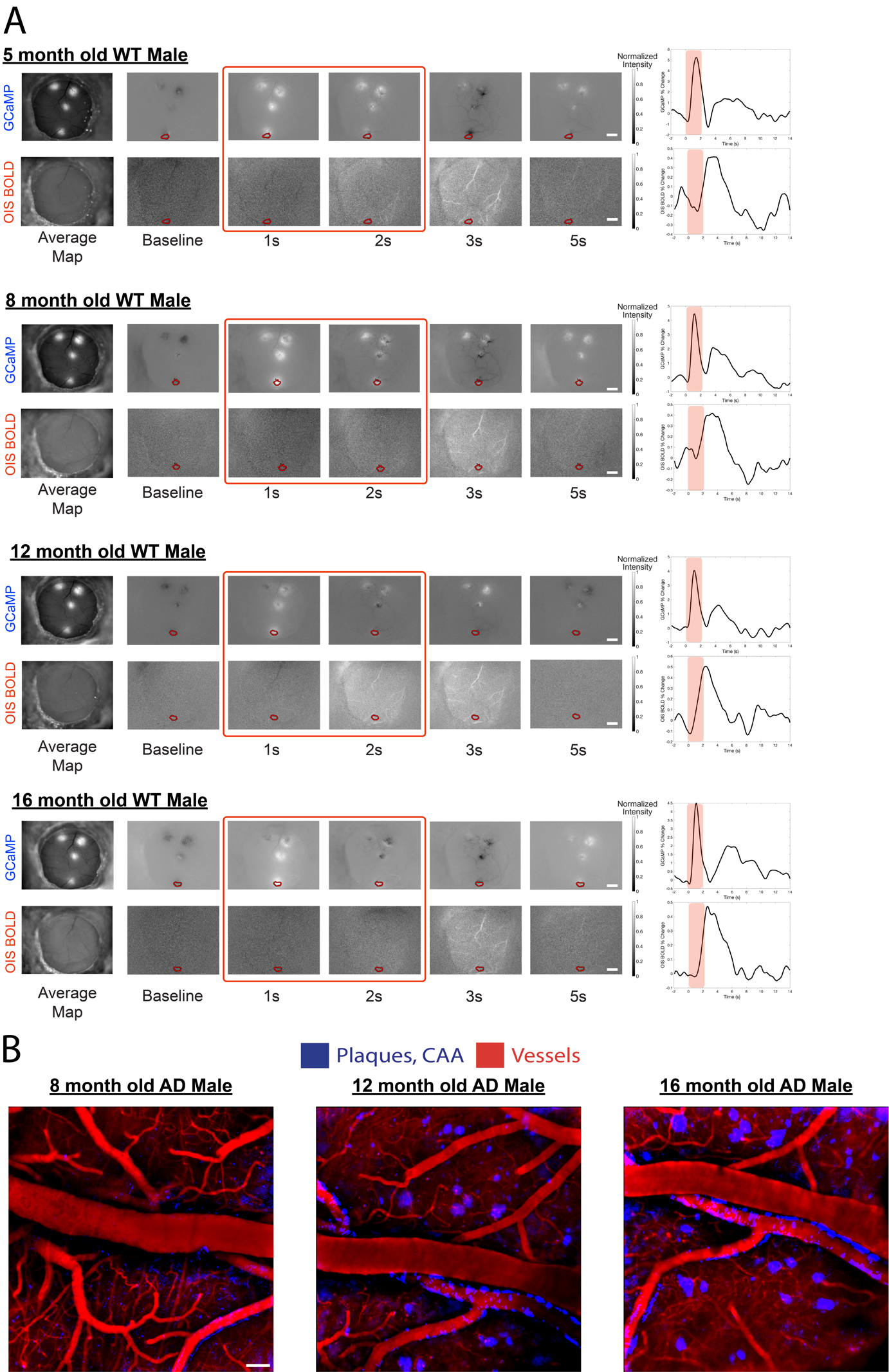


**Supplementary Figure 2. Representative longitudinal images from the two-photon and the widefield microscopy.** A.) Neurovascular coupling during whisker puff measured by GCaMP and OIS BOLD changes in the barrel cortex in the same wild type (WT) male mouse at 5, 8, 12 and 16 months of age (left column). The corresponding time series of fluorescent and OIS change presented in the right column were extracted from the activated whisker barrel (red region of interest). Scale bar = 500 um. B.) Longitudinal two-photon microscopy images in the same Alzheimer’s disease (AD) male mouse at 8, 12 and 16 months of age. Methoxy-04 labeled for amyloid-β (Aβ) in blue, blood vessels labeled with Sulforhodamine 101 (SR101) in red. Scale bar = 10 um.


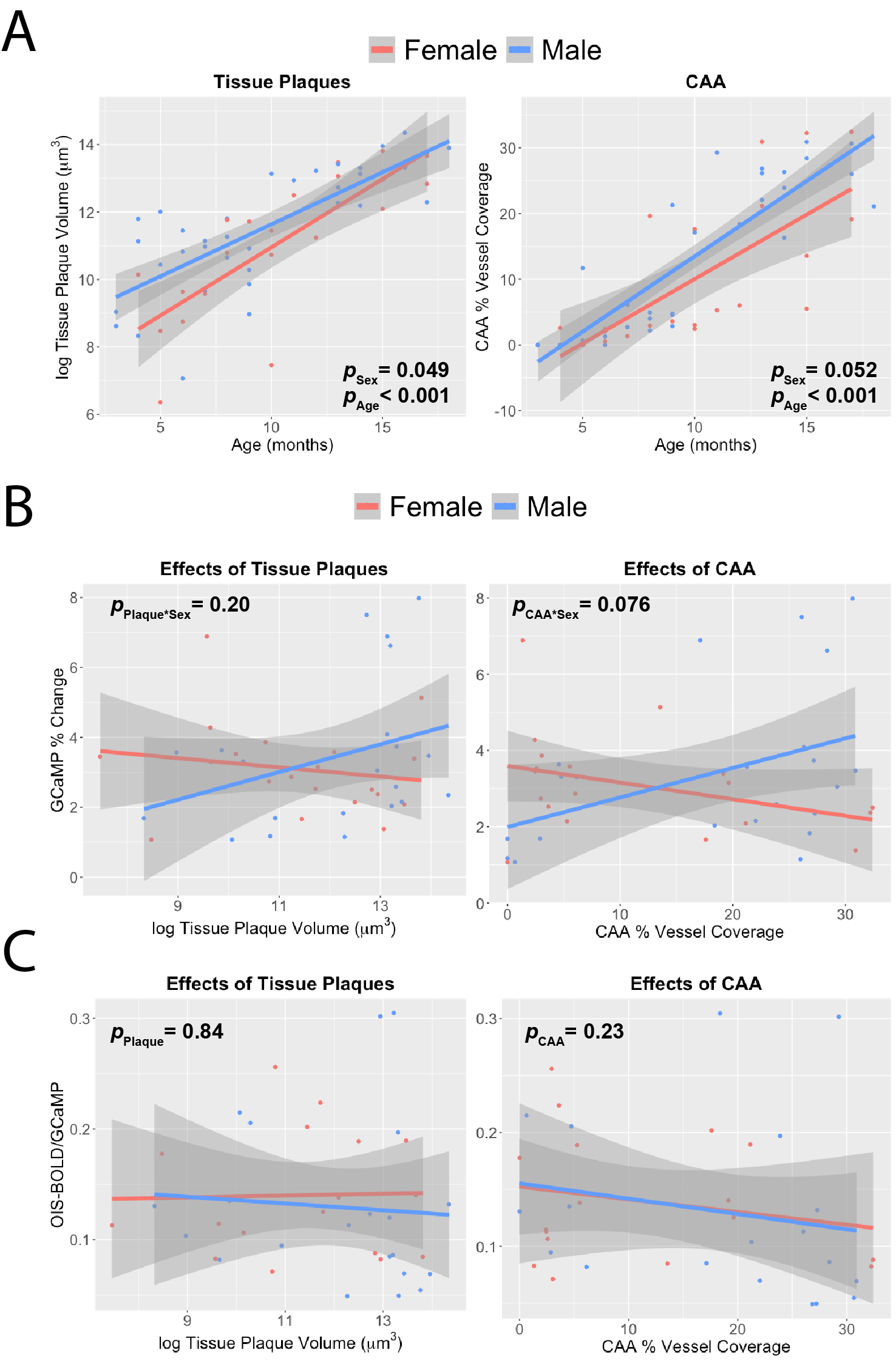


**Supplementary Figure 3. Neuronal response is associated with CAA in AD males.** A.) log tissue plaque volume (left) and CAA vessel coverage (right) versus age is displayed by each sex. Multivariate linear mixed effect regression was used to test the effect of age and sex on tissue plaque volume (*left panel*, *N* = 58) and CAA (*right panel*, *N* = 59). B.) The neuronal response is plotted by sex for each figure. The GCaMP response to whisker puffing was associated with CAA vessel coverage in AD males but not AD females. No association observed in either sex for tissue plaque volume. Multivariate linear mixed effect regression was used to test the interaction effect between age and sex on tissue plaque volume (*left panel*, *N* = 58) and CAA (*right panel*, *N* = 59). C.) The ratio of OIS BOLD/GCaMP was not associated with tissue plaque volume nor CAA vessel coverage in either sex. Multivariate linear mixed effect regression was used to test the effect of tissue plaque volume (*left panel*, *N* = 58) and CAA (*right panel*, *N* = 59) on the OIS BOLD/GCaMP ratio. In all panels, each data point represents a quantified measurement from a single imaging session for one mouse. Shaded area represents 95% confident interval. OIS-BOLD = deoxy-hemoglobin-weighted (blood oxygen level dependent) optical intrinsic signal; GCaMP = GCaMP6f fluorescent signal. CAA = Cerebral Amyloid Angiopathy.


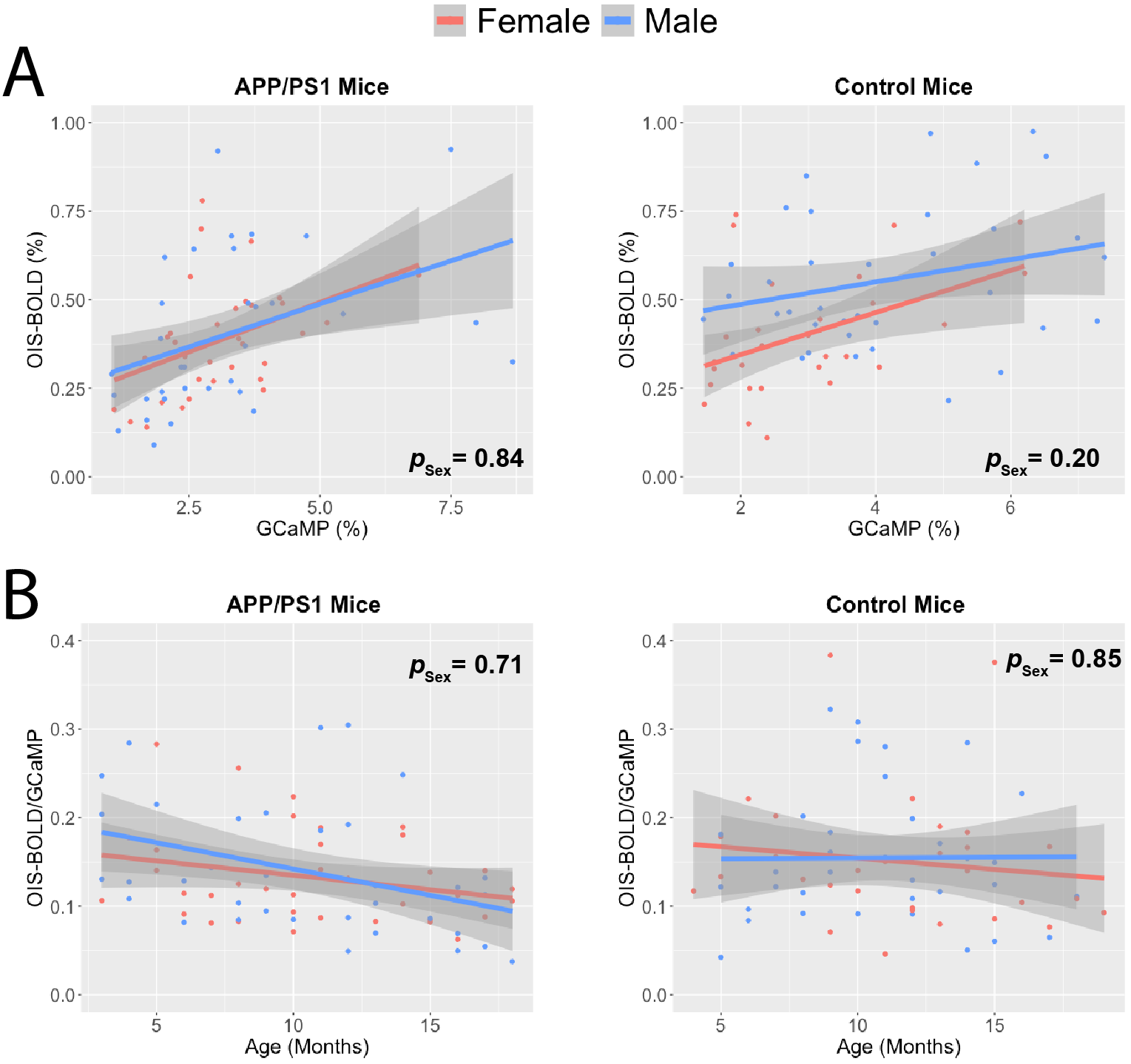


**Supplementary Figure 4. Neurovascular response does not show significant sex differences for AD mice, but control males have a higher vascular response with respect to neuronal response compared to control females.** A.) The vascular response versus the neuronal response is plotted by sex for each model. There were no significant sex differences for AD mice (left) but control males had a significantly higher OIS BOLD response compared to control females when controlling for GCaMP response (right). B.) No sex differences were observed for the ratio of OIS BOLD:GCaMP versus age in AD (left) or WT mice (right). In all panels, each data point represents a quantified measurement from a single imaging session for one mouse. Shaded area represents 95% confident interval. Multivariate linear mixed effect regression analysis was used in all figure panels to assess the effect of sex on OIS BOLD response while controlling for GCaMP response (N = 77 AD mice; N = 68 WT mice). OIS-BOLD = deoxy-hemoglobin-weighted (blood oxygen level dependent) optical intrinsic signal; CBV = total hemoglobin-weighted (cerebral blood volume) optical intrinsic signal; CAA = Cerebral Amyloid Angiopathy.

**
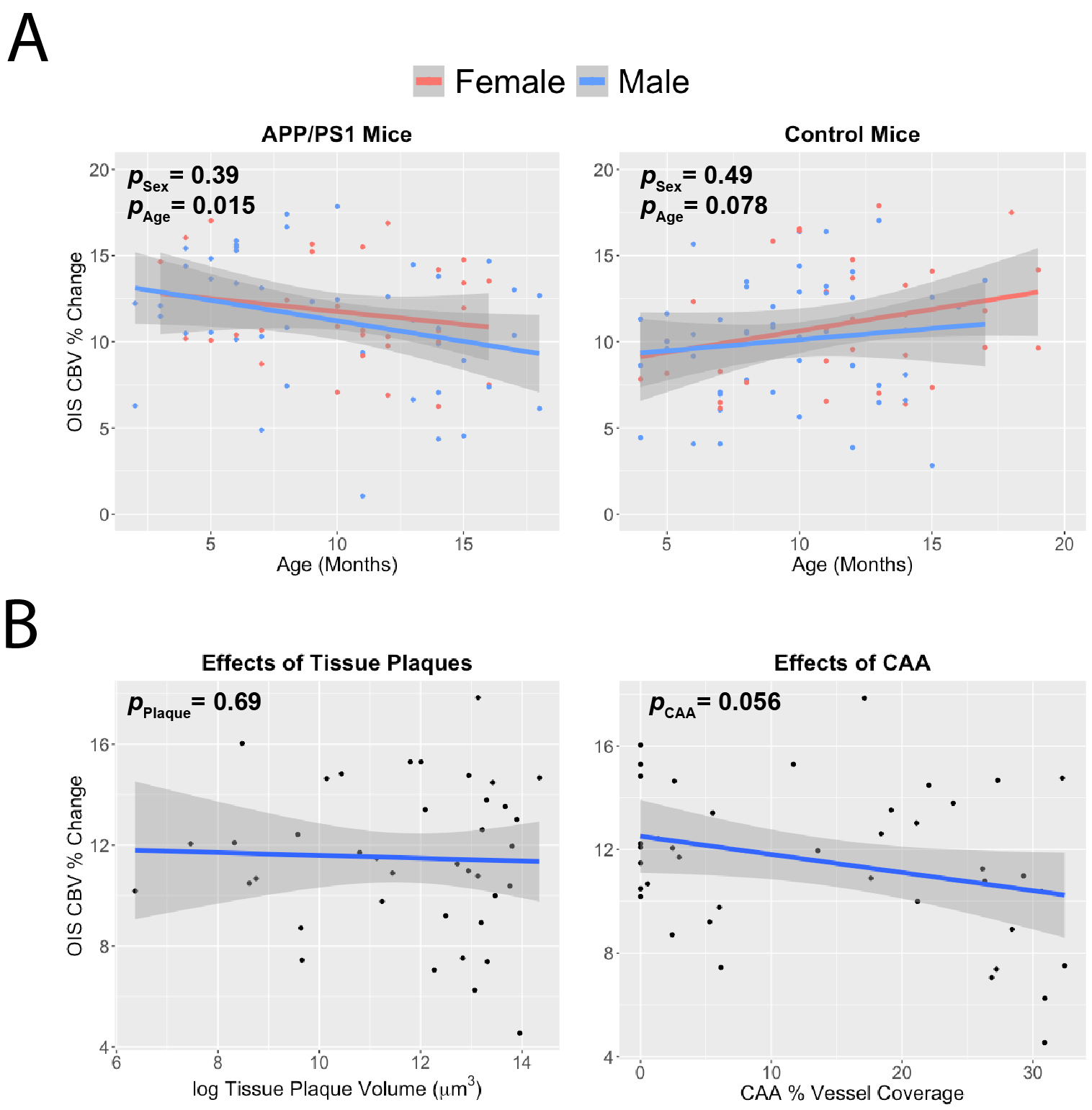
**

**Supplementary Figure 5. OIS CBV weighted response diminishes with age for AD mice but not control mice.** A.) CBV significantly decreases for AD mice (left) but not control mice (right) with age, and no sex differences were observed. Multivariate linear regression analysis was used to assess the effect of sex and age on OIS CBV response in AD mice (*left panel*, *N* = 108) and WT mice (*right panel*, *N* = 97). B.) The OIS CBV response was associated with CAA vessel coverage (right), but not Aβ plaque volume (left). Multivariate linear mixed effect regression analysis was used in all figure panels to assess the effect of tissue plaque volume and CAA on OIS CBV response while controlling for sex (*N* = 42). In all panels, each data point represents a quantified measurement from a single imaging session for one mouse. Shaded area represents 95% confident interval.


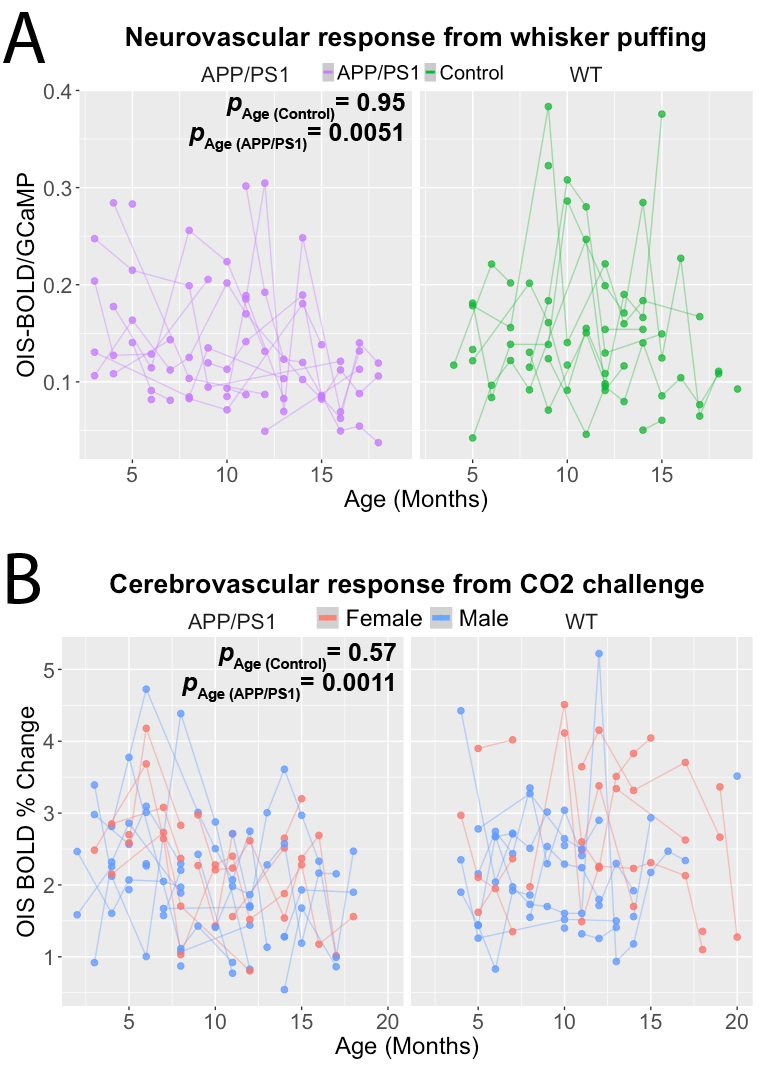


**Supplementary Figure 6. Individual longitudinal trajectories of neurovascular and cerebrovascular responses.** A.) Neurovascular response trajectories, measured as the ratio of OIS BOLD to GCaMP during whisker puff stimulation, are plotted for each individual mouse across age in AD (left, *N* = 77) and WT (right, *N* = 68) cohorts. Linear mixed effects modeling was used to assess the effect of age on the OIS BOLD:GCaMP ratio, with sex included as a fixed effect and a random effect to account for within-subject longitudinal variation. B.) Cerebrovascular response trajectories, measured as the OIS BOLD peak response during the CO_2_ challenge, are shown for AD mice (left, *N* = 108) and WT mice (right, *N* = 97) across age. The same linear mixed effects framework was applied to assess age effects, again controlling for sex and modeling random effects for each mouse. In all panels, individual data points represent measurements from a single imaging session, and each line traces the longitudinal trajectory of an individual mouse.
